# Foreign Plastid Sequences in Plant Mitochondria are Frequently Acquired Via Mitochondrion-to-Mitochondrion Horizontal Transfer

**DOI:** 10.1038/srep43402

**Published:** 2017-03-06

**Authors:** C. L. Gandini, M. V. Sanchez-Puerta

**Affiliations:** 1 IBAM, Universidad Nacional de Cuyo, CONICET, Facultad de Ciencias Agrarias, Almirante Brown 500, M5528AHB, Chacras de Coria, Argentina; 2 Facultad de Ciencias Exactas y Naturales, Universidad Nacional de Cuyo, 5500, Mendoza, Argentina

## Abstract

Angiosperm mitochondrial genomes (mtDNA) exhibit variable quantities of alien sequences. Many of these sequences are acquired by intracellular gene transfer (IGT) from the plastid. In addition, frequent events of horizontal gene transfer (HGT) between mitochondria of different species also contribute to their expanded genomes. In contrast, alien sequences are rarely found in plastid genomes. Most of the plant-to-plant HGT events involve mitochondrion-to-mitochondrion transfers. Occasionally, foreign sequences in mtDNAs are plastid-derived (MTPT), raising questions about their origin, frequency, and mechanism of transfer. The rising number of complete mtDNAs allowed us to address these questions. We identified 15 new foreign MTPTs, increasing significantly the number of those previously reported. One out of five of the angiosperm species analyzed contained at least one foreign MTPT, suggesting a remarkable frequency of HGT among plants. By analyzing the flanking regions of the foreign MTPTs, we found strong evidence for mt-to-mt transfers in 65% of the cases. We hypothesize that plastid sequences were initially acquired by the native mtDNA via IGT and then transferred to a distantly-related plant via mitochondrial HGT, rather than directly from a foreign plastid to the mitochondrial genome. Finally, we describe three novel putative cases of mitochondrial-derived sequences among angiosperm plastomes.

Since the endosymbiotic events that shaped the eukaryotic cells, cytoplasmic organelles - plastids and mitochondria - have transferred large part of their eubacterial genomes to the nucleus^1^. Today, DNA exchange between organelles and with the nuclear genome, known as intracellular gene transfer (IGT), continues to take place within plant cells at variable frequencies^2^,^3^. In addition, horizontal gene transfer (HGT), the genetic movement of DNA between unrelated species, is now accepted as a driving force in the evolution of land plants^4^. Flowering plants present exceptionally high rates of HGT, mainly involving the mitochondrial genome^5^,^6^. Plant mitochondrial genomes (mtDNA) commonly incorporate nuclear and plastid sequences acquired by IGT as well as foreign mitochondrial DNA from other plant species obtained by HGT.

Plastid-derived DNA is found in angiosperm mtDNAs (MTPTs) in variable amounts representing 0.1 to 10.3% of the mtDNAs and covering 0.5 to 87.2% of the plastid genomes^7^,^8^. Plastid-to-mitochondria transfers have been ongoing since the colonization of land plants^9^. Despite that most of the plastid-derived sequences result in non-functional sequences, it is now accepted that once integrated into the mitochondrial genome, MTPTs can impact mitochondrial function. For example, MTPTs can create new gene forms or promoters, or may introduce novel functional tRNA genes^10^,^11^,^12^,^13^. Interestingly, some MTPTs were acquired by HGT from distant angiosperm species^8^,^14^,^15^,^16^,^17^. Whether these sequences were acquired directly from the donor plastid or indirectly from the donor mitochondria is still unclear and it is the focus of the present study.

In contrast to mtDNAs, plastid genomes (ptDNAs) exhibit very low rates of alien DNA^18^. Lately, four mitochondrial-derived sequences located in angiosperm ptDNAs (PTMT) have been reported^19^,^20^,^21^,^22^. Here, we take advantage of the recent increase in plant organellar sequences in public databases to study the extent of MTPTs and PTMTs among flowering plants, and to weigh evidence on the genomic origin of foreign MTPTs.

## Results and Discussion

### MTPTs are invariably present in seed plants but are infrequent among non-seed plants

We analyzed the mitochondrial genomes of 136 diverse species of the green lineage and only identified MTPTs in gymnosperms (13 sequences) and angiosperms (1,372 sequences), and none among non-seed plants (Table S1). This is consistent with the ‘limited transfer window hypothesis’ that argues that species with a single plastid per cell, such as the majority of green algae, or species with monoplastidic meiosis, such as bryophytes and most lycophytes^23^, present less IGT events, if any, from the plastid to the nucleus or to the mitochondria^24^. Angiosperms showed the highest relative contents of MTPTs within the green lineage. *Geranium maderense* and *Phoenix dactylifera* ranked first with plastid-derived sequences covering 10.38% and 9.86% of their mtDNA, respectively (Table S1).

To evaluate the relationship between the size of the mtDNA and the MTPT content, we performed a Spearman non-parametric test (Figure S1). Interestingly, the size of the mitochondrial genome strongly correlates with the amount of plastid sequences in angiosperm and gymnosperm mtDNAs, considering the total MTPT length (rho = 0.57, P = 1.05 × 10^−07^) or the total number of MTPTs (rho = 0.64, P = 6.57 × 10^−10^), but not with the MTPT mitochondrial coverage (rho = 0.16, P = 0.1693). In general, larger mtDNAs give shelter to more MTPTs (Figure S1). This observation agrees with previous studies on MTPTs and also on organelle-to-nucleus DNA transfers^24^,^25^, suggesting that genomes with extensive non-coding regions could harbor more alien sequences, but these alien insertions are not solely responsible for plant mitochondrial genome expansion^26^.

### Foreign MTPTs are frequent among flowering plants

MTPTs can be derived from the plastid genome of the same species by IGT (termed native MTPTs) or from an unrelated species by HGT (termed foreign MTPTs). To determine the origin of the 1,385 MTPTs mentioned above (Table S1), all MTPTs with highest similarity to the ptDNA of an unrelated lineage were considered putatively foreign and were analyzed phylogenetically to confirm its origin and to determine the donor lineage. In addition to the 31 previously described cases^8^,^15^,^16^,^17^,^27^, 15 new foreign MTPTs were identified in this work (Table 1). MTPTs were considered foreign when phylogenetic analyses showed unexpected relationships with bootstrap support (BS) higher than 70% (Figure S2). In all cases, donor lineages were identified as members of the flowering plants, indicating angiosperm-to-angiosperm HGT events (Table 1, Figure S2). Out of the 72 angiosperm mtDNAs analyzed, 14 (19.4%) had at least one foreign MTPT (Table S1). That is, one out of five plant mtDNAs received plastid sequences by HGT. Sampling the >99.9% unexamined angiosperms may reveal that thousands of species bear foreign MTPTs in their mitochondria. These results are comparable to those of the *cox1* intron horizontal transfers among angiosperms, in which 20% of the sequenced species had the invasive *cox1* intron^28^. Among all alien DNA acquired by the mitochondrion, MTPTs and *cox1* introns have the highest probability of being detected as they carry strong phylogenetic signal to corroborate their foreign origin. Therefore, these markers could speak for the underlying rate of DNA transfer among angiosperms.

### Foreign MTPTs flanking regions strengthen the mt-to-mt transfer hypothesis

**Figure 1:**
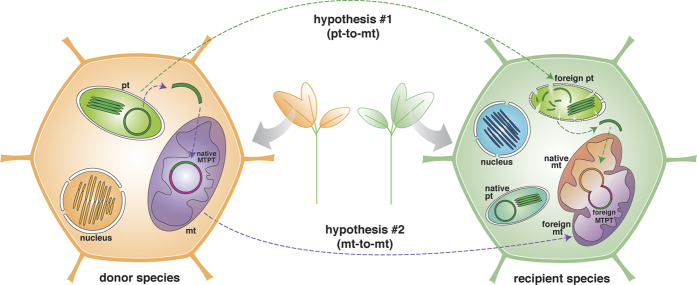
Hypotheses on the origin of foreign plastid sequences located in plant mtDNA (MTPTs). Hypothesis #1: Plant-to-plant interactions (direct contact or via vector intermediates) enable the transfer of entire plastids (pt) whose genomic sequences are freed into the recipient cell and then captured by the native mitochondria (mt). Hypothesis #2: Plastid sequences are transferred by intracellular gene transfer from the plastid to the mitochondria within the donor plant; later, plant-to-plant interactions enable the transfer of entire foreign mitochondria into the recipient cell, both mitochondria (foreign and native) fuse and their genomes recombine.

The identification of foreign plastid-derived sequences in plant mtDNAs raises questions about the trajectory taken by these sequences until their arrival into the mitochondria. Foreign MTPTs could have originated through horizontal transfer from two different sources: (i) directly from the foreign ptDNA; or (ii) indirectly from the foreign mtDNA once the latter acquired the plastid sequences by IGT (Fig. 1). We favor hypothesis #2 for the following reasons: (i) all angiosperm mtDNAs analyzed contain native MTPTs, indicating that the initial acquisition of the plastid sequence by the native mitochondria is a trivial event^8^,^24^; and (ii) relatively frequent mitochondrion-to-mitochondrion HGT events among plants have been reported^15^,^28^,^29^,^30^,^31^,^32^,^33^,^34^,^35^. Here, we searched for evidence to test this hypothesis by analyzing each MTPT in detail. We reasoned that under hypothesis #2, foreign MTPTs should be embedded within foreign mitochondrial tracts, which were transferred as a whole via mt-to-mt HGT. Assuming the mt-to-mt transfer, we expect that foreign flanking mitochondrial sequences will have the same origin, i.e. they are related to the same donor lineage, as the foreign MTPT.

We analyzed both flanking sequences (1 kb at each side) of the 46 foreign plastid insertions known to date (Table 1). BLAST and phylogenetic analyses of these regions revealed the presence of foreign mitochondrial sequences from the same donor lineage as the MTPT in 30 of the 46 cases (65%) (Table 1, Figure S3). Therefore, most of the foreign MTPTs were first integrated into the donor mitochondrial genome by IGT, and later horizontally transferred to the recipient mitochondria. The delivery of the MTPT from the donor mitochondria could follow the fusion-compatibility model^15^, in which the entire foreign mitochondria is captured by the recipient cell where the two mitochondria would fuse and their genomes recombine (Fig. 1).

Besides the 65% of the foreign MTPTs that showed evidence for mt-to-mt HGT, several MTPTs could not be fairly tested given the lack of mitochondrial genomic sequences from the donor lineages. For example, mitochondrial data from members of the family Fagaceae are not yet available, preventing the analyses of MTPTs found in *Amborella trichopoda* and *Phoenix dactylifera*. However, the upcoming sequencing of more plant mitochondrial genomes may uncover additional proof for the acquisition of other MTPTs via mitochondrial HGT.

Alternatively, a pt-to-mt horizontal transfer (hypothesis #1) is also conceivable. For example, it has been shown that plastids can be transferred through grafting between species^36^. Once in the recipient cell, the ptDNA can be freed and enter the mitochondria in the same way as the native ptDNA (Fig. 1). It is also possible that plastid DNA were horizontally transferred into the recipient cell and imported by the mitochondria^37^,^38^. Even though less likely than mt-to-mt HGT, pt-to-mt HGT may be responsible for some of the foreign MTPTs.

**Figure 2:**
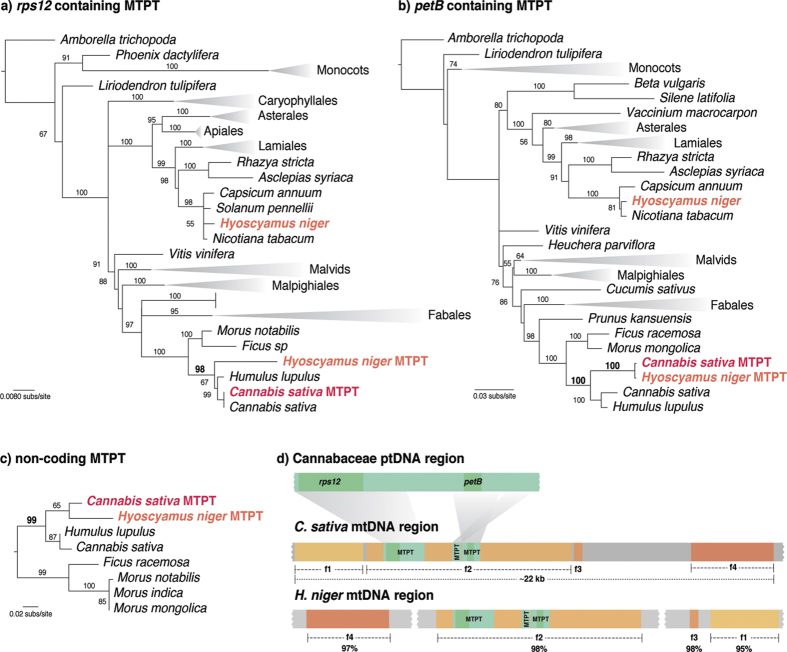
HGT from the family Cannabaceae to *Hyoscyamus niger* mtDNA. (**a–c**) Evidence of HGT from *Cannabis sativa* to *H. niger* mtDNA. Maximum likelihood trees of the plastid sequences *rps12* (**a**), *petB* (**b**), and a non-coding region (**c**) are shown and include sequences located in the plastid or mitochondrial (MTPT) genomes of angiosperms. Several branches are collapsed and shown as triangles for clarity; the full trees are shown in Figure S2. Bootstrap support values >50% are shown above the branches. (**d**) Plastid (ptDNA) and mitochondrial (mtDNA) genomic comparisons of *C. sativa* and *H. niger*. The percent identity between mitochondrial homologous regions (f1–f4) found in *C. sativa* and *H. niger* are shown below the *H. niger* mtDNA.

In only a few cases, mitochondrial genomic sequences were available from both donor and recipient lineages of the MTPTs, enabling more powerful comparisons. We found strong evidence for mt-to-mt HGT of foreign MTPTs in the angiosperm *Hyoscyamus niger* (Solanaceae). The three foreign plastid regions located in the *H. niger* mtDNA were confirmed to belong to the family *Cannabaceae* with strong phylogenetic support (BS ≥ 98%) (Fig. 2). Moreover, two of them, one containing the plastidial gene *petB* and the other a non-coding plastid region, were sister to the native MTPT found in *Cannabis sativa* mtDNA (Fig. 2b and c). The region containing the gene *rps12* showed a different genealogical history, given that the *C. sativa* MTPT was more closely related to the *C. sativa* plastome than to the *H. niger* MTPT (Fig. 2a). However, the three plastid-derived regions were embedded within a mitochondrial region of *C. sativa* mtDNA that is also present in the mtDNA of *H. niger* (f2 in Fig. 2d), pointing to a single mt-to-mt HGT event. Therefore, the most plausible scenario is that after the mt-to-mt HGT event from *C. sativa* to *H. niger*, a second pt-to-mt intracellular gene transfer was experienced by the *C. sativa rps12* MTPT^8^. To evaluate the extent of the mitochondrial HGT between those two species, we performed comparative analyses of both mitochondrial genomes. The analyses revealed the presence of four mitochondrial fragments (f1 to f4 in Fig. 2d) in *H. niger* mtDNA with high similarity (~95–98%) to sequences of *C. sativa* mtDNA (Fig. 2). Moreover, these mitochondrial sequences were only shared by *H. niger* and *C. sativa*. The four mitochondrial fragments, including the three MTPTs, were found within a 22 kb stretch of *C. sativa* mtDNA. This whole region was likely subjected to mt-to-mt transfer from a member of the family *Cannabaceae* to *H. niger* (Fig. 2d) and was slightly disrupted once integrated in the *H. niger* mtDNA.

**Figure 3:**
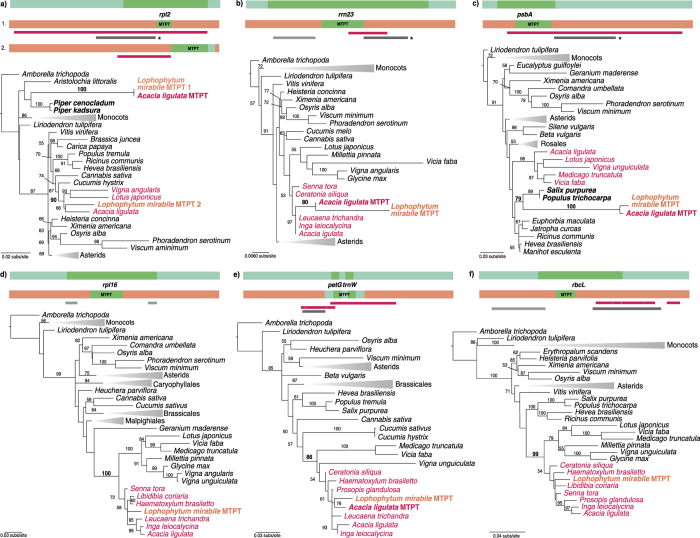
Analyses of foreign MTPTs in *Lophophytum mirabile* and their flanking regions. Green rectangles represent a fragment of the donor plastid genome depicting coding sequences in dark green. Orange rectangles represent fragments of the *L. mirabile* mtDNA denoting the MTPT in green. BLAST results of the flanking regions are indicated with lines in fuchsia (hits to *Acacia ligulata*; donor lineage), grey (hits unrelated to the donor or the recipient lineages), and dark grey (hits to diverse lineages). When hits to diverse lineages were found, they were aligned and analyzed by phylogenetic analyses (Figure S3). In most cases, sequences of *Acacia* mtDNA were sister to the flanking regions of *L. mirabile* (depicted with an *). Best ML trees of plastid and MTPT fragments are shown. Members of the Fabaceae are shown in fuchsia. Several branches are collapsed and shown as triangles for clarity; the full trees are shown in Figure S2. Bootstrap support values >50% are shown above the branches.

A remarkable number of HGT events among plants took place between hosts and parasites^16^,^17^,^27^,^31^,^32^,^34^. The haustorial connection that parasitic plants establish with their hosts provides a direct cell-to-cell contact, and a putative pathway for DNA transfers^39^. In agreement to this, we found that 24 of the 46 foreign MTPTs (52%) involved members of a parasitic relationship (shaded in grey in Table 1). Seven cases implicated the holoparasitic plant *Lophophytum mirabile*^27^ (Table 1, Fig. 3). Plants of the genus *Lophophytum* infect exclusively members of the tribe Mimosoideae (family Fabaceae)^40^ and phylogenetic analyses showed that five MTPTs of *L. mirabile* were acquired from its host^27^. However, two MTPTs were related to magnoliids and Salicales, respectively^27^. Here, we reanalyzed the MTPTs found in *L. mirabile* including recently available partial data from the mtDNA of the mimosoid *Acacia ligulata*^41^. Our results confirmed that five MTPTs were sister or nested within the tribe Mimosoideae (Fig. 3). In addition, the two MTPTs of *L. mirabile (rpl2* and *psbA*) with odd relationships were now found sister to MTPTs of *A. ligulata* mtDNA with high bootstrap support (Fig. 3, Figure S2). In those two cases, both plastid sequences found in *L. mirabile* and *A. ligulata* mtDNAs were misplaced in the tree and the direction of the transfer could not be inferred from these data. However, the unparalleled acquisition of mitochondrial sequences from the mimosoids by *L. mirabile*^27^ suggests that these were also the result of transfers from the host to the parasite. Under such assumption, *A. ligulata* mtDNA must have received plastid sequences from Piperales (*rpl2*) and Salicales (*rrn23*), respectively, before the HGT to *L. mirabile.* Blast and phylogenetic analyses showed that flanking sequences of five of the seven MTPTs of *L. mirabile* were only similar (e.g. *rpl2*) or highly related (e.g. *rrn23*) to *A. ligulata* mtDNA (Table 1, Figure S3). These findings support transfers from the mimosoids via mt-to-mt HGT for most MTPTs in *L. mirabile*. A deeper inspection of the complete sequence of the *A. ligulata* mtDNA should reveal the extent of the HGT in this host-parasite relationship.

### Mitochondrion-to-plastid DNA transfers are rare

In contrast to the universally present MTPTs in angiosperm mtDNAs, mitochondrial sequences in plastid genomes (PTMTs) are rare. Since the first PTMT described by *Iorizzo et al*.^19^ in the carrot ptDNA, only three more cases have been published for angiosperm plastids^20^,^21^,^22^ (Table S2). To evaluate the frequency of PTMTs in plant plastids, we analyzed a total of 1,232 land plant ptDNAs using BLAST (Table S3). In addition to the four cases already described, we found three further PTMTs (Table S2). Unfortunately, we cannot rule out assembly errors for these novel cases because the original reads were not available in the public databases or shared by the authors. Surprisingly, the PTMT found in the obligate root holoparasite *Orobanche californica* has 93% identity to a mitochondrial sequence of one of its various hosts, *Capsicum annuum*^42^, becoming the first putative case of HGT within a plastome (Table S2). Among the eight complete plastid genomes of non-photosynthetic parasites of the family Orobanchaceae^43^, *O. californica* is the only one that showed the aforementioned insertion in the ptDNA, suggesting a recent transfer event.

## Materials and Methods

To identify potential MTPTs we analyzed a total of 136 complete mitochondrial genomes of the green lineage (Table S1) available in the NCBI Organelle Genome Database as of April 2016 that have at least a plastid genome of the same order for comparison purposes. We blasted each mitochondrial genome against 1,232 plants plastid genomes available in the NCBI Organelle Genome Database using BLASTN v.2.4.0+ algorithm^44^ with the following settings: –task blastn –word_size 20 –e-value 1e-10. BLAST hits associated with ancient transfers^8^,^9^ or hits of ancient homology (*atp1, rrn18,* and *rrn26*)^12^ were excluded from further analysis. Mitochondrial sequences with blast hits to plastid genomes (named MTPTs) larger than 200 bp and with sequence identity >70% were further studied. MTPTs >200 bp separated by gaps <100 bp were taken together as one.

To detect MTPTs of foreign origin we searched for MTPTs with hits showing higher similarity to plastid sequences from a lineage unrelated to the one containing the MTPT. For each potential foreign MTPT, a set of homologous plastid sequences encompassing diverse plant species were extracted from NCBI databases and aligned using MUSCLE v3.7^45^. To confirm the identity of the donor lineage, Maximum Likelihood analyses (1,000 rapid bootstrapping replicates) under a GTR+G substitution model were performed with RAxML v.8.0.0^46^ (settings: -f a –m GTRGAMMA –k –N 1000 –x 67840 –p 7593029 –T 2). The presence of foreign MTPTs in the published mitochondrial assemblies was confirmed by paired-end read information, when available, also, in most cases, the library insert size was longer than the MTPTs. Flanking regions (1 kb at each side) of foreign MTPTs were analyzed with BLASTN using Unipro UGENE software^47^ to identify their origin. When BLAST hits included diverse angiosperms, we performed evolutionary analyses of the regions flanking the MTPT with RAxML, as described above.

To identify PTMTs we parsed a total of 1,232 complete plastid genomes that were available in the NCBI Organelle Genome Database as of August 2016 against all land plant mitochondrial genomes using BLASTN v.2.4.0+ algorithm^44^,^48^ with the following settings: –task blastn –word_size 7 –evalue 1e-10. For each hit, we fetched the subject mitochondrial features and excluded from further analyses all hits that were annotated as plastid-derived sequences or hits that held ancient homology between plastid and mitochondrial genomes (*atp1, rrn18,* and *rrn26*)^12^. The relevant regions were blasted against NCBI nr databases to corroborate their mitochondrial origin. We selected as potential PTMTs those sequences in which the bitscore value was higher for mitochondrial hits than for plastids.

## Additional Information

**How to cite this article:** Gandini, C. L. and Sanchez-Puerta, M. V. Foreign Plastid Sequences in Plant Mitochondria are Frequently Acquired Via Mitochondrion-to-Mitochondrion Horizontal Transfer. *Sci. Rep.* **7**, 43402; doi: 10.1038/srep43402 (2017).

**Publisher's note:** Springer Nature remains neutral with regard to jurisdictional claims in published maps and institutional affiliations.

## Supplementary Material

Supplementary Information

## Figures and Tables

**Table 1:** Information on foreign plastid sequences identified in flowering plant mitochondrial genomes.

# Foreign MTPT^a^	Recipient species^b^	Recipient lineage	mtDNA genBank accession number	Start (nt)	End (nt)	Length of MTPT (bp)	Gene content of MTPT	BS^c^ (%)	Putative donor lineage^d^	Evidence for mt-to-mt HGT in flanking regions of MTPT (+/−1000 bp)^e^	
										5′	3′
**1**	*Amborella trichopoda*^1^	basal Magnoliophyta; Amborellales	KF754801	124144	127171	3028	*psbC; psbD*	—	Santalales	no^1^	no^3^
**2**			KF754803	3077656	3078925	1270	*rbcL*	—	Santalales	**yes**^**5**^	no^3^
3				474251	476207	1957	*psaA*	—	rosids; fabids; Oxalidales	no^3^	no^5^
4			KF754799	57856	61814	3959	*rps7; rps12; trnV; rrnS*	—	rosids; fabids; Fagales; Fagaceae	**yes**^**5**^	no^3^
5	*Asclepias syriaca*	asterids; lamiids; Gentianales	NC_022796	29093	29432	340	*ndhB-1*	100	rosids; fabids; Fabales	**yes**^**4**^	no^1^
6			NC_022797	40124	42206	2083	*ndhB-1; rps7; rps12*	100	rosids; fabids; Fabales	no^1^	no^2^
7			NC_022798	310073	311353	1281	non-coding region	100	rosids; fabids; Fabales	no^3^	**yes**^**5**^
8			NC_022796	497713	497990	278	*ndhD*	100	rosids; malvids; Malvales; Malvaceae	no^1^	no^2^
**9**	*Cucurbita pepo*	rosids; fabids; Cucurbitales	NC_014050	252522	253546	1025	*cemA; petA*	100	asterids; lamiids; Lamiales; Orobanchaceae	no^1^	**yes**^**4**^
10			NC_014050	848217	849548	1332	*rps7*	93	rosids; fabids; Malpighiales; Euphorbiaceae; Acalyphoideae	no^2^	no^1^
11	*Erythranthe guttata*	asterids; lamiids; Lamiales	NC_018041	469539	469886	348	*rps7*	90	rosids; fabids; Fabales; Fabaceae	no^3^	no^3^
**12**	*Geranium brycei*^2^	rosids; malvids; Geraniales	KP974317	47781	49416	1636	*rbcL*	—	asterids; lamiids; Solanales; Convolvulaceae; Cuscuta	no^3^	no^3^
**13**			KP974313	55290	56417	1128	*psaA*	—		no^1^	**yes**^**5**^
**14**			KP974311	98551	100870	2320	*psaB; rps14*	—		no^3^	**yes**^**5**^
**15**			KP974317	42601	44151	1551	*atpB; atpE*	—		no^3^	no^3^
16			KP974311	343448	347166	3719	*rpl2 intron; rpoB 3'; rpoC1 exon 1; ndhJ*	—	rosids; fabids; Malpighiales; Euphorbiaceae; Acalyphoideae	no^3^	**yes**^**6**^
17			KP974311	87980	89010	1031	*petB*	—		**yes**^**6**^	**yes**^**6**^
18			KP974317	19534	20704	1171	*psbD*	—		no^1^	no^3^
19			KP974312	105427	106239	813	*matK*	—	asterids; lamiids; Gentianales; Rubiaceae; Rubioideae	no^3^	**yes**^**6**^
20	*Geranium maderense*	rosids; malvids; Geraniales	NC_027000	454189	454564	376	*trnA; trnG*	98	asterids; lamiids; Lamiales	no^3^	**yes**^**4**^
21	*Glycine max*^3^	rosids; fabids; Fabales	NC_020455	230204	230895	692	*rbcL*	—	asterids; lamiids; Gentianales; Apocynaceae	**yes**^**4**^	**yes**^**4**^
22	*Gossypium harknessii*	rosids; malvids; Malvales	NC_027406	365728	365950	223	*psbD*	82	rosids; fabids; Malpighiales; Euphorbiaceae	**yes**^**4**^	no^3^
	*Gossypium hirsutum*		NC_027407	368914	369136	223					
23	*Helianthus annuus*	asterids; campanulids; Asterales	NC_023337	107850	108495	646	*infA; rps8; rps11*	98	rosids; Saxifragales; Penthoraceae	no^5^	**yes**^**5**^
24	*Hyoscyamus niger*	asterids; lamiids; Solanales	NC_026515	351599	353572	1974	*rps12*	98	rosids; Rosales; Cannabaceae	**yes**^**4**^	**yes**^**4**^
25				354959	355152	194	non-coding region	99	rosids; Rosales; Cannabaceae^Ω^	**yes**^**4**^	**yes**^**4**^
26				355156	356418	1263	*petB*	100	rosids; Rosales; Cannabaceae; Cannabis^Ω^	**yes**^**4**^	**yes**^**4**^
**27**	*Lophophytum mirabile*^4^	Santalales	KU992322 to KU992380	—	—	245	*rpl2*	100	rosids; fabids; Fabales; Mimosoideae; Acacia ligulata^Ω^	**yes**^**5**^	**yes**^**4**^
**28**				—	—	726	*rrn23*	80	rosids; fabids; Fabales; Mimosoideae; Acacia ligulata^Ω^	no^3^	**yes**^**5**^
**29**				—	—	638	*psbA*	100	rosids; fabids; Fabales; Mimosoideae; Acacia ligulata^Ω^	**yes**^**4**^	**yes**^**5**^
**30**				—	—	520	*rpl16*	72	rosids; fabids; Fabales; Mimosoideae	no^3^	no^3^
**31**				—	—	673	*petG; trnW*	76	rosids; fabids; Fabales; Mimosoideae; Acacia ligulata^Ω^	**yes**^**5**^	**yes**^**4**^
**32**				—	—	269	*rbcL*	99	rosids; fabids; Fabales; Fabaceae	no^3^	no^5^
**33**				—	—	771	*rpl2*	90	rosids; fabids; Fabales; Fabaceae	**yes**^**4**^	no^1^
**34**	*Lotus japonicus*^3^	rosids; fabids; Fabales	NC_016743	307253	308275	1023	*rbcL*	—	asterids; lamiids; Solanales; Convolvulaceae; Cuscuta	**yes**^**4**^	no^3^
35	*Phoenyx dactylifera*^3^	Liliopsida; Arecaceae	NC_016740	179688	180534	847	*trnI; trnA*	—	rosids; fabids; Fagales; Fagaceae	no^3^	no^3^
36	*Rhazya stricta*	asterids; lamiids; Gentianales	NC_024293	236269	237891	1623	*trnI; ycf2*	87	asterids; lamiids; Lamiales; Oleaceae; Oleeae; Hesperelaea^Ω^	no^3^	**yes**^**4**^
37	*Salvia miltiorrhiza*	asterids; lamiids; Lamiales	NC_023209	209571	209920	350	*psbA*	100	rosids; fabids; Fabales; Fabaceae	**yes**^**4**^	**yes**^**5**^
**38**	*Sapria himalayana*^5^	rosids; fabids; Malpighiales	—	—	—	1737	*psbC; psbD*	—	rosids; Vitales; Tetrastigma^Ω^	no^1^	**yes**^**5**^
**39**			—	—	—	395	*psbA*	—	rosids; Vitales; Tetrastigma^Ω^	no^1^	no^3^
**40**			—	—	—	504	*ndhB*	—	rosids; Vitales; Tetrastigma	**yes**^**4**^	no^3^
**41**			—	—	—	477	*atpB*	—	rosids; Vitales; Tetrastigma^Ω^	no^1^	**yes**^**5**^
**42**			—	—	—	3703	*rpoC1; rpoC2*	—	rosids; Vitales; Tetrastigma	**yes**^**4**^	**yes**^**4**^
**43**			—	—	—	2595	*rps12*	—	rosids; Vitales	NA	**yes**^**5**^
**44**			—	—	—	436	*rbcL*	—	rosids; Vitales	no^3^	no^3^
**45**			—	—	—	360	*psaB*	—	rosids; Vitales; Tetrastigma^Ω^	no^1^	no^3^
**46**			—	—	—	457	*atpA*	—	asterids; campanulids; Apiales; Daucus	no^3^	no^1^

^a^Bold indicates MTPTs involving a host-parasite relationship.

^b^(1) Rice *et al*.^15^ (2) Park *et al*.^17^; (3) Sloan *et al*.^8^; (4) Sanchez-Puerta *et al*.^27^ and this study, Figure S2; (5) Xi *et al*.^16^.

^c^BS, bootstrap support value.

^d^Ω, the phylogenetic analysis showed the donor MTPT as sister to the MTPT of the recipient mitochondria.

^e^(1) no hit; (2) all hits related to the recipient lineage (putative native sequence); (3) hits to lineages unrelated to the donor or the recipient (unconclusive origin); (4) all hits related to the donor lineage; (5) hits to diverse lineages, phylogenetic analyses of flanking regions are shown in Figure S3; (6) Park *et al*.^17^; NA: not applicable, sequence not available for testing.
