# Supplementary material for: Foreign Plastid Sequences in Plant Mitochondria are Frequently Acquired Via Mitochondrion-to-Mitochondrion Horizontal Transfer
Source: Sci Rep. 2017 Mar 6;7:43402. doi: 10.1038/srep43402 (PMC5338292; doi:10.1038/srep43402)

Foreign plastid sequences in plant mitochondria are frequently acquired via mitochondrion-to-mitochondrion horizontal transfer

C.L. Gandini[1] and M.V. Sanchez-Puerta[1,2 *]

[1]IBAM, Universidad Nacional de Cuyo, CONICET, Facultad de Ciencias Agrarias, Almirante Brown 500, M5528AHB, Chacras de Coria, Argentina. [2]Facultad de Ciencias Exactas y Naturales, Universidad Nacional de Cuyo, 5500, Mendoza, Argentina.

*Author for Correspondence: M. Virginia Sanchez-Puerta
ORCID 0000-0003-2511-5093
Email: mvsanchezpuerta@fca.uncu.edu.ar
Phone +54 (261) 4135000 ext.1307

Table S1. Plastid-derived sequences in the mitochondrial genome of the green lineage

Species name	mtDNA GenBank accession number	ptDNA available?				MTPTs			mtDNA length (kb)	foreign MTPTs (> 200 bp)	
		from the same species	from the same genus	from the same family	from the same order	count (>200 bp)	total length (kb)	mt coverage (%)		count	total length (kb)
**Angiosperms (polyplastidic cells)**											
Aegilops speltoides	NC_022666	yes	-	-	-	14	15.13	3.18	476.09	-	-
Ajuga reptans	NC_023103	yes	-	-	-	5	2.33	0.66	352.07	-	-
Allium cepa	NC_024813	yes	-	-	-	16	27.95	8.84	316.36	-	-
Amborella trichopoda	KF754799-KF754803	yes	-	-	-	61	130.32	3.37	3866.04	4	10.21
Arabidopsis thaliana	NC_001284	yes	-	-	-	9	4.85	1.32	366.92	-	-
Asclepias syriaca	NC_022796	yes	-	-	-	41	53.08	7.78	682.50	4	3.98
Batis maritima	NC_024429	no	no	no	yes	9	3.38	0.84	403.93	-	-
Beta macrocarpa	NC_015994	no	yes	-	-	10	8.73	2.26	385.22	-	-
Beta vulgaris subsp. maritima	NC_015099	no	yes	-	-	17	7.87	2.16	364.95	-	-
Beta vulgaris subsp. vulgaris	NC_002511	yes	-	-	-	17	8.56	2.32	368.80	-	-
Boea hygrometrica	NC_016741	yes	-	-	-	35	44.72	8.76	510.52	-	-
Brassica carinata	NC_016120	no	yes	-	-	7	6.26	2.69	232.24	-	-
Brassica juncea	NC_016123	yes	-	-	-	6	7.13	3.24	219.77	-	-
Brassica napus	NC_008285	yes	-	-	-	5	5.25	2.36	221.85	-	-
Brassica nigra	NC_029182	yes	-	-	-	6	6.23	2.68	232.41	-	-
Brassica oleracea var. oleracea	NC_016118	no	yes	-	-	16	14.28	3.96	360.27	-	-
Brassica rapa	NC_016125	yes	-	-	-	6	7.12	3.24	219.75	-	-
Cannabis sativa	NC_029855	yes	-	-	-	13	6.69	1.61	415.60	-	-
Capsicum annuum	NC_024624	yes	-	-	-	28	46.70	9.13	511.53	-	-
Carica papaya	NC_012116	yes	-	-	-	14	22.91	4.80	476.89	-	-
Citrullus lanatus	NC_014043	no	no	yes	-	14	14.59	3.85	379.24	-	-
Cucumis sativus	NC_016004-NC_016006	yes	-	-	-	27	63.48	4.77	1331.31	-	-
Cucurbita pepo	NC_014050	no	no	yes	-	41	74.77	7.61	982.83	2	2.06
Daucus carota	NC_017855	yes	-	-	-	9	8.61	3.06	281.13	-	-
Erythranthe guttata	NC_018041	no	yes	-	-	15	15.97	3.04	525.67	1	0.35
Geranium maderense	NC_027000	yes	-	-	-	53	76.48	10.38	737.09	1	0.38
Glycine max	NC_020455	yes	-	-	-	15	5.30	1.32	402.56	1	0.69
Gossypium barbadense	NC_028254	yes	-	-	-	22	11.94	1.76	677.43	-	-
Gossypium harknessii	NC_027407	no	yes	-	-	22	10.68	1.60	666.08	1	0.22
Gossypium hirsutum	NC_027406	yes	-	-	-	21	11.61	1.74	668.58		
Gossypium raimondii	NC_029998	yes	-	-	-	15	9.19	1.36	676.08	-	-
Helianthus annuus	NC_023337	yes	-	-	-	12	6.73	2.24	300.95	1	0.60
Heuchera parviflora	KR559021	yes	-	-	-	10	11.53	2.12	542.95	-	-
Hevea brasiliensis	AP014526	yes	-	-	-	22	29.31	2.21	1325.82	-	-
Hyoscyamus niger	NC_026515	yes	-	-	-	23	13.70	2.73	501.40	2	2.96
Liriodendron tulipifera	NC_021152	yes	-	-	-	14	27.17	4.91	553.72	-	-
Lophophytum mirabile	KU992322-KU992380	no	no	no	yes	7	3.84	2.20	821.92	7	3.84
Lotus japonicus	NC_016743	yes	-	-	-	13	7.38	1.94	380.86	1	1.02
Malus domestica	NC_018554	no	yes	-	-	7	2.82	0.71	396.95	-	-
Medicago truncatula	NC_029641	no	yes	-	-	8	2.78	1.02	271.62	-	-
Millettia pinnata	NC_016742	yes	-	-	-	7	4.41	1.03	425.72	-	-
Nicotiana tabacum	NC_006581	yes	-	-	-	14	11.86	2.75	430.60	-	-
Oryza minuta	NC_029816	yes	-	-	-	36	38.79	7.53	515.02	-	-
Oryza rufipogon	NC_013816	yes	-	-	-	29	31.81	5.69	559.05	-	-
Oryza sativa Indica group	NC_007886	yes	-	-	-	47	34.82	7.08	491.52	-	-
Oryza sativa Japonica group	NC_011033	yes	-	-	-	47	33.98	6.93	490.52	-	-
Phoenix dactylifera	NC_016740	yes	-	-	-	30	70.49	9.86	715.00	1	0.85
Populus tremula	NC_028096	yes	-	-	-	53	19.97	2.55	783.00	-	-
Populus tremula x Populus alba	NC_028329	yes	-	-	-	53	20.39	2.60	783.00	-	-
Raphanus sativus	NC_018551	yes	-	-	-	12	9.94	3.85	258.43	-	-
Rhazya stricta	NC_024293	yes	-	-	-	17	31.11	5.67	548.61	1	1.62
Salix purpurea	NC_029693	yes	-	-	-	20	19.65	3.28	598.97	-	-
Salix suchowensis	NC_029317	yes	-	-	-	20	14.87	2.31	644.44	-	-
Salvia miltiorrhiza	NC_023209	yes	-	-	-	17	16.73	3.35	499.24	1	0.35
Silene latifolia	NC_014487	yes	-	-	-	5	2.10	0.83	253.41	-	-
Silene noctiflora	JF750431-JF750489	yes	-	-	-	8	2.48	0.04	6727.87	-	-
Silene vulgaris	JF750427-JF750430	yes	-	-	-	7	9.74	2.28	427.14	-	-
Sorghum bicolor	NC_008360	yes	-	-	-	17	26.67	5.69	468.63	-	-
Spirodela polyrhiza	NC_017840	yes	-	-	-	11	7.65	3.35	228.49	-	-
Tripsacum dactyloides	NC_008362	no	no	yes	-	21	25.41	3.61	704.10	-	-
Triticum aestivum	NC_007579	yes	-	-	-	27	19.89	4.40	452.53	-	-
Triticum timopheevii	NC_022714	yes	-	-	-	12	12.83	2.89	443.42	-	-
Vaccinium macrocarpon	NC_023338	yes	-	-	-	16	7.27	1.58	459.68	-	-
Vigna angularis	NC_021092	yes	-	-	-	5	3.04	0.75	404.47	-	-
Vigna radiata	NC_015121	yes	-	-	-	7	3.25	0.81	401.26	-	-
Viscum album	NC_029039	yes	-	-	-	2	0.41	0.07	565.00	-	-
Vitis vinifera	NC_012119	yes	-	-	-	22	58.45	7.56	773.28	-	-
Zea luxurians	NC_008333	yes	-	-	-	14	19.58	3.63	539.37	-	-
Zea mays subsp. mays	NC_007982	yes	-	-	-	27	6.48	1.14	569.63	-	-
Zea mays subsp. parviglumis	NC_008332	yes	-	-	-	27	16.83	2.47	680.60	-	-
Zea perennis	NC_008331	yes	-	-	-	17	20.87	3.66	570.35	-	-
Ziziphus jujuba	NC_029809	yes	-	-	-	16	22.35	6.12	365.20	-	-
**SubTotal**	**72**					**1372**				**28**	

**Gymnosperms (polyplastidic cells)**											
Cycas taitungensis	NC_010303	yes	-	-	-	6	13.28	3.20	414.90	-	-
Ginkgo biloba	NC_027976	yes	-	-	-	2	0.87	0.25	346.54	-	-
Welwitschia mirabilis	NC_029130	yes	-	-	-	5	6.65	0.68	978.85	-	-
**SubTotal**	**3**					**13**				**0**	
**Mosses (polyplastidic cells, monoplastidic cells during meiosis)**											
Anomodon attenuatus	NC_021931	no	no	no	yes	-	-	-	104.25	-	-
Anomodon rugelii	NC_016121	no	no	no	yes	-	-	-	104.24	-	-
Climacium americanum	NC_024515	no	no	no	yes	-	-	-	105.05	-	-
Hypnum imponens	NC_024516	no	no	no	yes	-	-	-	103.83	-	-
Orthotrichum diaphanum	NC_029356	no	yes	-	-	-	-	-	104.74	-	-
Orthotrichum macrocephalum	NC_029355	no	yes	-	-	-	-	-	104.62	-	-
Orthotrichum speciosum	NC_026121	no	yes	-	-	-	-	-	104.75	-	-
Orthotrichum stellatum	NC_024522	no	yes	-	-	-	-	-	104.13	-	-
Physcomitrella patens	NC_007945	yes	-	-	-	-	-	-	105.34	-	-
**SubTotal**	**9**					**0**				**0**	
**Lycopods (monoplastidic cells)**											
Huperzia squarrosa	NC_017755	no	yes	-	-	-	-	-	413.53	-	-
**SubTotal**	**1**					**0**				**0**	
**Liverworts (monoplastidic cells)**											
Aneura pinguis	NC_026901	no	yes	-	-	-	-	-	165.60	-	-
Marchantia polymorpha	NC_001660	yes	-	-	-	-	-	-	186.61	-	-
**SubTotal**	**2**					**0**				**0**	
**Hornworts (monoplastidic cells)**											
Oltmannsiellopsis viridis	NC_008256	yes	-	-	-	-	-	-	56.76	-	-
**SubTotal**	**1**					**0**				**0**	
**Green Algae (most of them monoplastidic cells)**											
**Charophycean green algae**											
Chaetosphaeridium globosum	NC_004118	yes	-	-	-	-	-	-	56.57	-	-
Chara vulgaris	NC_005255	yes	-	-	-	-	-	-	67.74	-	-
Chlorokybus atmophyticus	NC_009630	yes	-	-	-	-	-	-	201.76	-	-
Closterium baillyanum	NC_022860	no	no	no	yes	-	-	-	152.09	-	-
Entransia fimbriata	NC_022861	yes	-	-	-	-	-	-	61.65	-	-
Klebsormidium flaccidum	DF238763	yes	-	-	-	-	-	-	106.47	-	-
**SubTotal**	**6**					**0**				**0**	
**Division Chlorophyta**											
Auxenochlorella protothecoides	NC_026009	yes	-	-	-	-	-	-	57.27	-	-
Bathycoccus prasinos	NC_023273	yes	-	-	-	-	-	-	43.61	-	-
Botryococcus braunii	NC_027722	yes	-	-	-	-	-	-	84.58	-	-
Bracteacoccus aerius	NC_024755	yes	-	-	-	-	-	-	47.16	-	-
Bracteacoccus minor	NC_024756	yes	-	-	-	-	-	-	45.18	-	-
Chlamydomonas leiostraca	NC_026573	no	yes	-	-	-	-	-	14.03	-	-
Chlamydomonas moewusii	NC_001872	no	yes	-	-	-	-	-	22.90	-	-
Chlamydomonas reinhardtii	NC_001638	yes	-	-	-	-	-	-	15.76	-	-
Chlorella sorokiniana	NC_024626	yes	-	-	-	-	-	-	52.53	-	-
Chlorella variabilis	NC_025413	yes	-	-	-	-	-	-	78.50	-	-
Chlorotetraedron incus	NC_024757	yes	-	-	-	-	-	-	38.41	-	-
Chromochloris zofingiensis	NC_024758	yes	-	-	-	-	-	-	44.84	-	-
Coccomyxa sp	NC_015316	yes	-	-	-	-	-	-	65.50	-	-
Dunaliella salina	NC_012930	yes	-	-	-	-	-	-	28.33	-	-
Dunaliella viridis	NC_026571	no	yes	-	-	-	-	-	45.98	-	-
Gonium pectorale	NC_020437	no	no	no	yes	-	-	-	15.99	-	-
Helicosporidium sp	NC_017841	yes	-	-	-	-	-	-	49.34	-	-
Kirchneriella aperta	NC_024759	yes	-	-	-	-	-	-	52.93	-	-
Lobosphaera incisa	NC_027060	yes	-	-	-	-	-	-	70.00	-	-
Micromonas sp	NC_012643	yes	-	-	-	-	-	-	47.43	-	-
Microspora stagnorum	NC_022862	no	no	yes	-	-	-	-	87.41	-	-
Monomastix sp	NC_022797	yes	-	-	-	-	-	-	60.88	-	-
Monoraphidium neglectum	NW_014013625	yes	-	-	-	-	-	-	93.84	-	-
Mychonastes homosphaera	NC_024760	yes	-	-	-	-	-	-	25.15	-	-
Neochloris aquatica	NC_024761	yes	-	-	-	-	-	-	38.02	-	-
Nephroselmis olivacea	NC_008239	yes	-	-	-	-	-	-	45.22	-	-
Ostreococcus tauri	NC_008290	yes	-	-	-	-	-	-	44.24	-	-
Ourococcus multisporus	NC_024762	no	yes	-	-	-	-	-	49.71	-	-
Pedinomonas minor	NC_000892	yes	-	-	-	-	-	-	25.14	-	-
Pleodorina starrii	NC_021108	yes	-	-	-	-	-	-	20.38	-	-
Polytoma uvella	NC_026572	no	no	no	yes	-	-	-	17.41	-	-
Polytomella capuana	NC_010357	no	no	no	yes	-	-	-	13.00	-	-
Polytomella magna	NC_023091	no	no	no	yes	-	-	-	24.37	-	-
Polytomella parva	NC_016916	no	no	no	yes	-	-	-	13.14	-	-
Polytomella parva	NC_016917	no	no	no	yes	-	-	-	3.02	-	-
Polytomella sp	NC_013472	no	no	no	yes	-	-	-	13.00	-	-
Polytomella sp	NC_016918	no	no	no	yes	-	-	-	3.08	-	-
Prasinoderma coloniale	NC_023355	yes	-	-	-	-	-	-	54.55	-	-
Prototheca wickerhamii	NC_001613	no	no	yes	-	-	-	-	55.33	-	-
Pseudendoclonium akinetum	NC_005926	yes	-	-	-	-	-	-	95.88	-	-
Pseudomuriella schumacherensis	NC_024763	yes	-	-	-	-	-	-	43.13	-	-
Pycnococcus provasolii	NC_013935	yes	-	-	-	-	-	-	24.32	-	-
**SubTotal**	**42**					**0**				**0**	
**TOTAL**	**136**					**1385**				**28**	

Table S2. Mitochondrial sequences identified in flowering plant plastid genomes.

# PTMT	recipient plastid genome		ptDNA GenBank accession number	PTMT				putative donor of the mitochondrial sequence		Identity (%)
	species[a]	lineage		start (nt)	end (nt)	length (bp)	gene content	species	lineage	
1	*Daucus carota* [1]	Asterids; campanulids; Apiales; Apiaceae	NC_008325	99297	100748	1452	partial *coxI*	*Daucus carota*	Asterids; campanulids; Apiales; Apiaceae	99
2	*Asclepias spp.*[2]	Asterids; lamiids; Gentianales; Apocynaceae	NC_022432	16591	19225	2635	non-coding region	*Asclepias syriaca*	Asterids; lamiids; Gentianales; Apocynaceae	85
3	*Pariana spp.*[3]	Poales; Poaceae; BOP clade	NC_027491; KP319245; KP319246	84561	87686	3126	non-coding region	*Ferrocalamus rimosivaginus*	Poales; Poaceae; BOP clade	98
4	*Paspalum spp.*[4]	Poales; Poaceae; PACMAD clade	NC_030495	84665	86100	1436	non-coding region	*Tripsacum dactyloides*	Poales; Poaceae; PACMAD clade	98
				86867	87405	539				99
5	*Lavandula angustifolia* [5]	Asterids; lamiids; Lamiales; Lamiaceae	NC_029370	152063	153448	1386	*trnH-GUG*	*Salvia miltiorhizza*	Asterids; lamiids; Lamiales; Lamiaceae	95
6	*Orobanche californica* [5]	Asterids; lamiids; Lamiales; Orobanchaceae	NC_025651	87733	88413	681	orf144, hypothetical protein	*Capsicum annuum*	Asterids; lamiids; Solanales; Solanaceae	93
7	*Scutellaria baicalensis* [5]	Asterids; lamiids; Lamiales; Lamiaceae	NC_027262	152335	152731	397	*trnH-GUG*	*Mimulus guttatus*	Asterids; lamiids; Lamiales; Phrymaceae	96

[a] 1) Iorizzo et al, 2012; 2) Straub et al, 2013; 3) Ma et al, 2015; 4) Burke et al, 2016; 5) this study

Table S3. List of complete plastid genomes analyzed in this study.

NC_026892 Abies koreana; NC_026134 Acacia ligulata; NC_030331 Acer davidii; NC_030343 Acer miaotaiense; NC_029371 Acer morrisonense; NC_015820 Acidosasa purpurea; NC_030534 Acioa guianensis; NC_030056 Acnistus arborescens; NC_030185 Acnistus arborescens; NC_029973 Acoelorraphe wrightii; NC_029829 Aconitum chiisanense; NC_010093 Acorus americanus; NC_007407 Acorus calamus; NC_026299 Acorus gramineus; NC_026690 Actinidia chinensis; NC_026691 Actinidia deliciosa; NC_008101 Acutodesmus obliquus; NC_026999 Adenophora remotiflora; NC_004766 Adiantum capillus; NC_024831 Aegilops bicornis; NC_023096 Aegilops cylindrica; NC_023097 Aegilops geniculata; NC_024832 Aegilops kotschyi; NC_024830 Aegilops longissima; NC_024815 Aegilops searsii; NC_024816 Aegilops sharonensis; NC_022135 Aegilops speltoides; NC_022133 Aegilops tauschii; NC_009265 Aethionema cordifolium; NC_009266 Aethionema grandiflorum; NC_030544 Afrolicania elaeosperma; NC_023119 Agathis dammara; NC_015621 Ageratina adenophora; NC_023357 Agrostemma githago; NC_008591 Agrostis stolonifera; NC_023102 Ajuga reptans; NC_029427 Akebia trifoliata; NC_024813 Allium cepa; NC_022413 Allosyncarpia ternata; NC_027951 Alloteropsis angusta; NC_027952 Alloteropsis cimicina; NC_027824 Alloteropsis semialata; NC_012818 Alsophila spinulosa; NC_005086 Amborella trichopoda; NC_027581 Amentotaxus argotaenia; NC_024945 Amentotaxus formosana; NC_027465 Ammophila breviligulata; NC_024731 Ampelocalamus calcareus; NC_027466 Ampelodesmos mauritanicus; NC_030619 Amphicarpum muhlenbergianum; NC_026220 Ananas comosus; NC_022451 Andrographis paniculata; NC_029470 Anethum graveolens; NC_010359 Aneura mirabilis; NC_030545 Angelesia splendens; NC_029391 Angelica acutiloba; NC_029392 Angelica dahurica; NC_029393 Angelica gigas; NC_026300 Angiopteris angustifolia; NC_008829 Angiopteris evecta; NC_022412 Angophora costata; NC_022411 Angophora floribunda; NC_029735 Ankyra judayi; NC_030166 Annona cherimola; NC_014062 Anomochloa marantoidea; NC_004543 Anthoceros formosae; NC_027467 Anthoxanthum odoratum; NC_015113 Anthriscus cerefolium; NC_029243 Aquilaria sinensis; NC_030346 Arabidopsis arenicola; NC_029334 Arabidopsis arenosa; NC_029335 Arabidopsis cebennensis; NC_030347 Arabidopsis croatica; NC_030348 Arabidopsis neglecta; NC_029336 Arabidopsis pedemontana; NC_030349 Arabidopsis petrogena; NC_030350 Arabidopsis suecica; NC_000932 Arabidopsis thaliana; NC_030351 Arabidopsis umezawana; NC_023367 Arabis alpina; NC_009268 Arabis hirsuta; NC_022810 Aralia undulata; NC_026450 Araucaria heterophylla; NC_021121 Ardisia polysticta; NC_029972 Areca vestiaria; NC_029971 Arenga caudata; NC_025228 Aristida purpurea; NC_020607 Artemisia frigida; NC_025910 Artemisia montana; NC_030613 Arthraxon prionodes; NC_023934 Arundinaria appalachiana; NC_024712 Arundinaria fargesii; NC_020341 Arundinaria gigantea; NC_023935 Arundinaria tecta; NC_030620 Arundinella deppeana; NC_022431 Asclepias nivea; NC_022432 Asclepias syriaca; NC_027434 Aster spathulifolius; NC_029828 Astragalus mongholicus; NC_028171 Astragalus nakaianus; NC_029891 Astrebla pectinata; NC_004561 Atropa belladonna; NC_030546 Atuna racemosa; NC_023775 Auxenochlorella protothecoides; NC_027468 Avena sativa; NC_030501 Axonopus fissifolius; NC_023792 Azadirachta indica; NC_026958 Bambusa arnhemica; NC_026957 Bambusa bambos; NC_015830 Bambusa emeiensis; NC_024668 Bambusa multiplex; NC_012927 Bambusa oldhamii; NC_009269 Barbarea verna; NC_024811 Bathycoccus prasinos; NC_029970 Baxteria australis; NC_030062 Berberis amurensis; NC_022457 Berberis bealei; NC_030063 Berberis koreana; NC_020366 Bismarckia nobilis; NC_029483 Bletilla ochracea; NC_028422 Bletilla striata; NC_016468 Boea hygrometrica; NC_025306 Bomarea edulis; NC_029969 Borassodendron machadonis; NC_029420 Boswellia sacra; NC_030621 Bothriochloa alta; NC_025545 Botryococcus braunii; NC_025641 Boulardia latisquama; NC_029414 Bouteloua curtipendula; NC_029892 Bouteloua gracilis; NC_026036 Bowenia serrulata; NC_027470 Brachyelytrum aristosum; NC_011032 Brachypodium distachyon; NC_029675 Bracteacoccus aerius; NC_028586 Bracteacoccus giganteus; NC_029674 Bracteacoccus minor; NC_029968 Brahea brandegeei; NC_022811 Brassaiopsis hainla; NC_028272 Brassica juncea; NC_016734 Brassica napus; NC_030450 Brassica nigra; NC_015139 Brassica rapa; NC_028633 Brighamia insignis; NC_027471 Briza maxima; NC_027472 Bromus vulgaris; NC_030180 Bruinsmia polysperma; NC_013359 Bryopsis

hypnoides; NC_026795 Bryopsis plumosa; NC_026968 Buergersiochloa bambusoides; NC_027834 Bupleurum falcatum; NC_009599 Buxus microphylla; NC_020365 Calamus caryotoides; NC_024544 Calanthe triplicata; NC_026295 Callitropsis nootkatensis; NC_026298 Callitropsis vietnamensis; NC_023121 Calocedrus formosana; NC_004993 Calycanthus floridus; NC_029337 Camelina sativa; NC_024541 Camellia crapnelliana; NC_022459 Camellia cuspidata; NC_022460 Camellia danzaiensis; NC_024659 Camellia grandibracteata; NC_022461 Camellia impressinervis; NC_024660 Camellia leptophylla; NC_023084 Camellia oleifera; NC_024661 Camellia petelotii; NC_022462 Camellia pitardii; NC_024662 Camellia pubicosta; NC_024663 Camellia reticulata; NC_020019 Camellia sinensis; NC_022264 Camellia taliensis; NC_022463 Camellia yunnanensis; NC_026203 Campanula takesimana; NC_026785 Campynema lineare; NC_026562 Cannabis sativa; NC_030622 Capillipedium venustum; NC_009270 Capsella bursa; NC_028517 Capsella grandiflora; NC_027693 Capsella rubella; NC_018552 Capsicum annuum; NC_030543 Capsicum chinense; NC_028007 Capsicum frutescens; NC_026551 Capsicum lycianthoides; NC_026445 Cardamine impatiens; NC_026446 Cardamine resedifolia; NC_027250 Carex siderosticta; NC_010323 Carica papaya; NC_026786 Carludovica palmata; NC_027618 Carnegiea gigantea; NC_028585 Carteria cerasiformis; NC_029889 Carum carvi; NC_029948 Caryota mitis; NC_014674 Castanea mollissima; NC_023801 Castanopsis echinocarpa; NC_021423 Catharanthus roseus; NC_014589 Cathaya argyrophylla; NC_026568 Cattleya crispata; NC_014575 Cedrus deodara; NC_024171 Cenchrus americanus; NC_024286 Centaurea diffusa; NC_025229 Centotheca lappacea; NC_029411 Centropodia glauca; NC_021110 Cephalotaxus oliveri; NC_016063 Cephalotaxus wilsoniana; NC_009962 Ceratophyllum demersum; NC_026037 Ceratozamia hildae; NC_004115 Chaetosphaeridium globosum; NC_029967 Chamaerops humilis; NC_008097 Chara vulgaris; NC_028584 Characiochloris acuminata; NC_014592 Cheilanthes lindheimeri; NC_027184 Chikusichloa aquatica; NC_024714 Chimonocalamus longiusculus; NC_025230 Chionochloa macra; NC_005353 Chlamydomonas reinhardtii; NC_026565 Chloranthus japonicus; NC_009598 Chloranthus spicatus; NC_023835 Chlorella sorokiniana; NC_015359 Chlorella variabilis; NC_001865 Chlorella vulgaris; NC_029893 Chloris barbata; NC_008822 Chlorokybus atmophyticus; NC_029673 Chlorotetraedron incus; NC_025539 Choricystis parasitica; NC_029672 Chromochloris zofingiensis; NC_020320 Chrysanthemum indicum; NC_020092 Chrysanthemum x; NC_024061 Chrysobalanus icaco; NC_029884 Chrysopogon serrulatus; NC_029966 Chuniophoenix nana; NC_027490 Chusquea circinata; NC_026969 Chusquea liebmannii; NC_026959 Chusquea spectabilis; NC_011163 Cicer arietinum; NC_021111 Cistanche deserticola; NC_025642 Cistanche phelypaea; NC_024929 Citrus aurantiifolia; NC_030194 Citrus platymamma; NC_008334 Citrus sinensis; NC_028000 Clematis terniflora; NC_030314 Closterium baillyanum; NC_015084 Coccomyxa sp; NC_029253 Cochlearia borzaeana; NC_029254 Cochlearia islandica; NC_029331 Cochlearia pyrenaica; NC_029332 Cochlearia tridactylites; NC_022417 Cocos nucifera; NC_008535 Coffea arabica; NC_030053 Coffea canephora; NC_013273 Coix lacryma; NC_030064 Colchicum autumnale; NC_025231 Coleataenia prionitis; NC_030358 Coleochaete scutata; NC_028080 Colobanthus quitensis; NC_016753 Colocasia esculenta; NC_028026 Colpothrinax cookii; NC_023131 Conopholis americana; NC_025659 Corallorhiza bulbosa; NC_025660 Corallorhiza macrantha; NC_025661 Corallorhiza mertensiana; NC_025664 Corallorhiza odontorhiza; NC_025662 Corallorhiza trifida; NC_025663 Corallorhiza wisteriana; NC_029850 Coriandrum sativum; NC_022409 Corymbia eximia; NC_022407 Corymbia gummifera; NC_028409 Corymbia henryi; NC_022408 Corymbia maculata; NC_022410 Corymbia tessellaris; NC_028410 Corymbia torelliana; NC_014807 Corynocarpus laevigata; NC_029965 Corypha lecomtei; NC_030357 Cosmarium botrytis; NC_030547 Couepia caryophylloides; NC_030548 Couepia grandiflora; NC_024063 Couepia guianensis; NC_030549 Couepia ovalifolia; NC_030550 Couepia paraensis; NC_030551 Couepia polyandra; NC_030552 Couepia rankiniae; NC_030553 Couepia sandwithii; NC_030554 Couepia subcordata; NC_009271 Crucihimalaya wallichii; NC_010548 Cryptomeria japonica; NC_023544 Cucumis hystrix; NC_015983 Cucumis melo; NC_007144 Cucumis sativus; NC_021437 Cunninghamia lanceolata; NC_028155 Cupressus gigantea; NC_026296 Cupressus sempervirens; NC_028729 Curcuma flaviflora; NC_022928 Curcuma roscoeana; NC_009963 Cuscuta exaltata; NC_009765 Cuscuta gronovii; NC_009949 Cuscuta obtusiflora; NC_009766 Cuscuta reflexa; NC_020319 Cycas revoluta; NC_009618 Cycas taitungensis; NC_030359

Cylindrocystis brebissonii; NC_021429 Cymbidium aloifolium; NC_028525 Cymbidium ensifolium; NC_027743 Cymbidium faberi; NC_028524 Cymbidium goeringii; NC_029711 Cymbidium kanran; NC_029712 Cymbidium lancifolium; NC_029713 Cymbidium macrorhizon; NC_021433 Cymbidium mannii; NC_021430 Cymbidium sinense; NC_021431 Cymbidium tortisepalum; NC_021432 Cymbidium tracyanum; NC_030169 Cymbomonas tetramitiformis; NC_029460 Cynanchum auriculatum; NC_029459 Cynanchum wilfordii; NC_028005 Cynara baetica; NC_028006 Cynara cornigera; NC_027113 Cynara humilis; NC_026772 Cypripedium formosanum; NC_027227 Cypripedium japonicum; NC_024421 Cypripedium macranthos; NC_028542 Cyrtomium devexiscapulae; NC_028705 Cyrtomium falcatum; NC_030555 Dactyladenia bellayana; NC_030556 Dactyladenia buchneri; NC_030557 Dactyladenia floretii; NC_027473 Dactylis glomerata; NC_025232 Danthonia californica; NC_030502 Danthoniopsis dinteri; NC_020367 Dasypogon bromeliifolius; NC_018117 Datura stramonium; NC_008325 Daucus carota; NC_028549 Dendrobium chrysotoxum; NC_028430 Dendrobium huoshanense; NC_029456 Dendrobium nobile; NC_024019 Dendrobium officinale; NC_029705 Dendrobium pendulum; NC_027691 Dendrobium strongylanthum; NC_013088 Dendrocalamus latiflorus; NC_026546 Dendropanax dentiger; NC_027607 Dendropanax morbifer; NC_023533 Deschampsia antarctica; NC_026960 Diandrolyra sp; NC_027474 Diarrhena obovata; NC_030623 Dichanthelium acuminatum; NC_025546 Dicloster acuatus; NC_025524 Dictyochloropsis reticulata; NC_027272 Dieffenbachia seguine; NC_024176 Digitaria exilis; NC_027512 Dioon spinulosum; NC_009601 Dioscorea elephantipes; NC_024170 Dioscorea rotundata; NC_027090 Dioscorea zingiberensis; NC_029750 Diplopanax stachyanthus; NC_024158 Diplopterygium glaucum; NC_029338 Dipteronia sinensis; NC_029894 Distichlis bajaensis; NC_029895 Distichlis spicata; NC_009272 Draba nemorosa; NC_008456 Drimys granadensis; NC_029770 Drosera rotundifolia; NC_026906 Dunalia brachyacantha; NC_026563 Dunalia obovata; NC_027099 Dunalia solanacea; NC_016732 Dunaliella salina; NC_028719 Echinochloa crus; NC_029827 Echinochloa frumentacea; NC_024643 Echinochloa oryzicola; NC_025655 Echites umbellatus; NC_028066 Elaeagnus macrophylla; NC_017602 Elaeis guineensis; NC_030486 Eleusine indica; NC_016430 Eleutherococcus senticosus; NC_027266 Elleanthus sodiroi; NC_025548 Elliptochloris bilobata; NC_018541 Elodea canadensis; NC_025233 Elytrophorus spicatus; NC_027514 Encephalartos lehmannii; NC_030313 Entransia fimbriata; NC_011954 Ephedra equisetina; NC_029347 Ephedra foeminea; NC_001568 Epifagus virginiana; NC_029941 Epimedium acuminatum; NC_029942 Epimedium dolichostemon; NC_029943 Epimedium koreanum; NC_029944 Epimedium lishihchenii; NC_029945 Epimedium pseudowushanense; NC_029428 Epimedium sagittatum; NC_026449 Epipogium aphyllum; NC_026448 Epipogium roseum; NC_027954 Epipremnum aureum; NC_014699 Equisetum arvense; NC_020146 Equisetum hyemale; NC_029412 Eragrostis minor; NC_029413 Eragrostis tef; NC_029964 Eremospatha macrocarpa; NC_025234 Eriachne stipacea; NC_030624 Eriochloa meyeriana; NC_029882 Eriochrysis cf; NC_029883 Eriochrysis laxa; NC_030171 Eriolarynx fasciculata; NC_026847 Erodium absinthoides; NC_015083 Erodium carvifolium; NC_027065 Erodium chrysanthum; NC_025906 Erodium crassifolium; NC_025907 Erodium gruinum; NC_014569 Erodium texanum; NC_024635 Erodium trifolium; NC_018114 Erycina pusilla; NC_030212 Erythranthe lutea; NC_030601 Erythroxylum novogranatense; NC_025532 Ettlia pseudoalveolaris; NC_022396 Eucalyptus aromaphloia; NC_022382 Eucalyptus baxteri; NC_022398 Eucalyptus camaldulensis; NC_022394 Eucalyptus cladocalyx; NC_022388 Eucalyptus cloeziana; NC_022391 Eucalyptus curtisii; NC_022399 Eucalyptus deglupta; NC_022380 Eucalyptus delegatensis; NC_022402 Eucalyptus diversicolor; NC_022383 Eucalyptus diversifolia; NC_022385 Eucalyptus elata; NC_022406 Eucalyptus erythrocorys; NC_008115 Eucalyptus globulus; NC_014570 Eucalyptus grandis; NC_022405 Eucalyptus guilfoylei; NC_022390 Eucalyptus marginata; NC_022392 Eucalyptus melliodora; NC_022404 Eucalyptus microcorys; NC_022395 Eucalyptus nitens; NC_022378 Eucalyptus obliqua; NC_022389 Eucalyptus patens; NC_022393 Eucalyptus polybractea; NC_022379 Eucalyptus radiata; NC_022386 Eucalyptus regnans; NC_022397 Eucalyptus saligna; NC_022403 Eucalyptus salmonophloia; NC_022384 Eucalyptus sieberi; NC_022400 Eucalyptus spathulata; NC_022401 Eucalyptus torquata; NC_022387 Eucalyptus umbra; NC_022381 Eucalyptus verrucata; NC_029963 Eugeissona tristis; NC_027744 Eugenia uniflora; NC_030503 Eulalia aurea; NC_028067 Euonymus japonicus;

NC_029429 Euptelea pleiosperma; NC_025305 Eustrephus latifolius; NC_029379 Eutrema botschantzevii; NC_029378 Eutrema halophilum; NC_028728 Eutrema heterophyllum; NC_028170 Eutrema salsugineum; NC_028727 Eutrema yunnanense; NC_030558 Exellodendron barbatum; NC_010776 Fagopyrum esculentum; NC_027161 Fagopyrum tataricum; NC_024715 Fargesia nitida; NC_024716 Fargesia spathacea; NC_024717 Fargesia yunnanensis; NC_027685 Fatsia japonica; NC_015831 Ferrocalamus rimosivaginus; NC_019648 Festuca altissima; NC_011713 Festuca arundinacea; NC_019649 Festuca ovina; NC_019650 Festuca pratensis; NC_028185 Ficus racemosa; NC_014346 Floydiella terrestris; NC_029469 Foeniculum vulgare; NC_019601 Fragaria chiloensis; NC_024258 Fragaria iinumae; NC_018767 Fragaria mandshurica; NC_015206 Fragaria vesca; NC_018766 Fragaria vesca; NC_019602 Fragaria virginiana; NC_021101 Francoa sonchifolia; NC_024728 Fritillaria cirrhosa; NC_024736 Fritillaria hupehensis; NC_023247 Fritillaria taipaiensis; NC_025543 Fusochloris perforata; NC_024718 Gaoligongshania megalothyrsa; NC_030559 Gaulettia elata; NC_024719 Gelidocalamus tessellatus; NC_025544 Geminella minor; NC_025652 Genlisea margaretae; NC_027442 Gentiana crassicaulis; NC_027441 Gentiana straminea; NC_030319 Gentiana tibetica; NC_030045 Geranium incanum; NC_029999 Geranium maderense; NC_014573 Geranium palmatum; NC_016986 Ginkgo biloba; NC_025538 Gloeotilopsis sterilis; NC_030065 Gloriosa superba; NC_021647 Glycine canescens; NC_021645 Glycine cyrtoloba; NC_021648 Glycine dolichocarpa; NC_021649 Glycine falcata; NC_030329 Glycine gracilis; NC_007942 Glycine max; NC_022868 Glycine soja; NC_021646 Glycine stenophita; NC_021650 Glycine syndetika; NC_021636 Glycine tomentella; NC_024038 Glycyrrhiza glabra; NC_026301 Gnetum gnemon; NC_021438 Gnetum montanum; NC_011942 Gnetum parvifolium; NC_028734 Gnetum ula; NC_020438 Gonium pectorale; NC_026773 Goodyera fumata; NC_029363 Goodyera procera; NC_029364 Goodyera schlechtendaliana; NC_029365 Goodyera velutina; NC_023213 Gossypium anomalum; NC_016712 Gossypium arboreum; NC_018112 Gossypium areysianum; NC_008641 Gossypium barbadense; NC_023214 Gossypium bickii; NC_018111 Gossypium capitis; NC_016670 Gossypium darwinii; NC_017894 Gossypium gossypioides; NC_016692 Gossypium herbaceum; NC_023215 Gossypium herbaceum; NC_007944 Gossypium hirsutum; NC_018109 Gossypium incanum; NC_023216 Gossypium longicalyx; NC_016711 Gossypium mustelinum; NC_016668 Gossypium raimondii; NC_018113 Gossypium robinsonii; NC_018110 Gossypium somalense; NC_023217 Gossypium stocksii; NC_023218 Gossypium sturtianum; NC_015204 Gossypium thurberi; NC_016690 Gossypium tomentosum; NC_026835 Gossypium turneri; NC_030560 Grangeria borbonica; NC_026961 Greslania sp; NC_029749 Guadua angustifolia; NC_029232 Guadua chacoensis; NC_026991 Guadua weberbaueri; NC_010601 Guizotia abyssinica; NC_030061 Gymnospermium microrrhynchum; NC_028614 Gynochthodes nanlingensis; NC_029484 Gynostemma pentaphyllum; NC_026775 Habenaria pantlingiana; NC_028583 Hafniomonas laevis; NC_025235 Hakonechloa macra; NC_027668 Haloxylon ammodendron; NC_027669 Haloxylon persicum; NC_024732 Hanabusaya asiatica; NC_029962 Hanguana malayana; NC_029819 Haplostachys haplostachya; NC_007977 Helianthus annuus; NC_030275 Helianthus argophyllus; NC_030173 Helianthus debilis; NC_023110 Helianthus decapetalus; NC_023109 Helianthus divaricatus; NC_023107 Helianthus giganteus; NC_023108 Helianthus grosseserratus; NC_023111 Helianthus hirsutus; NC_023114 Helianthus maximiliani; NC_023113 Helianthus strumosus; NC_023112 Helianthus tuberosus; NC_020362 Heliconia collinsiana; NC_008100 Helicosporidium sp; NC_027469 Helictochloa hookeri; NC_027159 Heloniopsis tubiflora; NC_025787 Hesperelaea palmeri; NC_026297 Hesperocyparis glabra; NC_015308 Hevea brasiliensis; NC_026909 Hibiscus syriacus; NC_026962 Hickelia madagascariensis; NC_027475 Hierochloe odorata; NC_029415 Hilaria cenchroides; NC_029896 Hilaria rigida; NC_030561 Hirtella macrosepala; NC_024066 Hirtella physophora; NC_024060 Hirtella racemosa; NC_030562 Hirtella suffulta; NC_030563 Hirtella zanzibarica; NC_027476 Hordeum jubatum; NC_008590 Hordeum vulgare; NC_028032 Humulus lupulus; NC_030564 Hunga gerontogea; NC_006861 Huperzia lucidula; NC_029358 Hydnora visseri; NC_030625 Hyparrhenia subplumosa; NC_023260 Hypseocharis bilobata; NC_009600 Illicium oligandrum; NC_030487 Imperata cylindrica; NC_015803 Indocalamus longiauritus; NC_024720 Indocalamus wilsonii; NC_024721 Indosasa sinica; NC_028732 Inga leiocalycina; NC_025542 Interfilum terricola; NC_029833 Iochroma australe; NC_029746 Iochroma cardenasianum; NC_030178

Iochroma cyaneum; NC_030177 Iochroma ellipticum; NC_030167 Iochroma lehmannii; NC_026726 Iochroma loxense; NC_026567 Iochroma nitidum; NC_030168 Iochroma salpoanum; NC_026574 Iochroma stenanthum; NC_027177 Iochroma tingoanum; NC_030044 Iochroma umbellatum; NC_029333 Ionopsidium acaule; NC_026703 Ipomoea batatas; NC_009808 Ipomoea purpurea; NC_024936 Iris gatesii; NC_029227 Iris sanguinea; NC_025236 Isachne distichophylla; NC_028415 Isatis tinctoria; NC_030488 Ischaemum afrum; NC_030611 Iseilema macratherum; NC_014675 Isoetes flaccida; NC_015543 Jacobaea vulgaris; NC_008407 Jasminum nudiflorum; NC_012224 Jatropha curcas; NC_028582 Jenufa minuta; NC_028581 Jenufa perforata; NC_028617 Juglans regia; NC_024021 Juniperus bermudiana; NC_028190 Juniperus cedrus; NC_024022 Juniperus monosperma; NC_024023 Juniperus scopulorum; NC_024024 Juniperus virginiana; NC_022814 Kalopanax septemlobus; NC_011930 Keteleeria davidiana; NC_029676 Kirchneriella aperta; NC_024167 Klebsormidium flaccidum; NC_025536 Koliella corcontica; NC_025531 Koliella longiseta; NC_029874 Kolkwitzia amabilis; NC_030565 Kostermanthus robustus; NC_007578 Lactuca sativa; NC_029808 Lagerstroemia fauriei; NC_029885 Lagerstroemia guilinensis; NC_030484 Lagerstroemia indica; NC_016058 Larix decidua; NC_028023 Larrea tridentata; NC_027838 Lathraea squamaria; NC_027148 Lathyrus clymenum; NC_027073 Lathyrus davidii; NC_027074 Lathyrus graminifolius; NC_027149 Lathyrus inconspicuus; NC_027075 Lathyrus japonicus; NC_027076 Lathyrus littoralis; NC_027077 Lathyrus ochroleucus; NC_027150 Lathyrus odoratus; NC_027078 Lathyrus palustris; NC_027079 Lathyrus pubescens; NC_014063 Lathyrus sativus; NC_027151 Lathyrus tingitanus; NC_027080 Lathyrus venosus; NC_029370 Lavandula angustifolia; NC_024106 Lecomtella madagascariensis; NC_016677 Leersia tisserantii; NC_030482 Leitneria floridana; NC_010109 Lemna minor; NC_027152 Lens culinaris; NC_027835 Leontopodium leiolepis; NC_009273 Lepidium virginicum; NC_027513 Lepidozamia peroffskyana; NC_009681 Leptosira terrestris; NC_028733 Leucaena trichandra; NC_029961 Leucothrinax morrisii; NC_024064 Licania alba; NC_030566 Licania canescens; NC_030567 Licania glabriflora; NC_024062 Licania heteromorpha; NC_030568 Licania macrophylla; NC_030569 Licania majuscula; NC_030570 Licania membranacea; NC_030571 Licania michauxii; NC_030572 Licania micrantha; NC_030573 Licania minutiflora; NC_030574 Licania ovalifolia; NC_024065 Licania sprucei; NC_030575 Licania tomentosa; NC_029394 Ligusticum tenuissimum; NC_029937 Lilium distichum; NC_027674 Lilium hansonii; NC_027679 Lilium sp; NC_026787 Lilium superbum; NC_027675 Lilium tsingtauense; NC_022859 Lindenbergia philippensis; NC_023092 Liquidambar formosana; NC_030504 Liriodendron chinense; NC_008326 Liriodendron tulipifera; NC_026970 Lithachne pauciflora; NC_026577 Lithocarpus balansae; NC_025533 Lobosphaera incisa; NC_009274 Lobularia maritima; NC_029960 Lodoicea maldivica; NC_019651 Lolium multiflorum; NC_009950 Lolium perenne; NC_026839 Lonicera japonica; NC_002694 Lotus japonicus; NC_030612 Loudetiopsis kerstingii; NC_030540 Ludisia discolor; NC_023090 Lupinus luteus; NC_025333 Luzuriaga radicans; NC_022136 Lygodium japonicum; NC_026197 Lysimachia coreana; NC_025288 Macadamia integrifolia; NC_028074 Machilus balansae; NC_028073 Machilus yunnanensis; NC_027511 Macrozamia mountperriensis; NC_030576 Magnistipula butayei; NC_023234 Magnolia cathcartii; NC_018357 Magnolia denudata; NC_020318 Magnolia grandiflora; NC_023237 Magnolia kobus; NC_015892 Magnolia kwangsiensis; NC_023238 Magnolia liliifera; NC_020316 Magnolia officinalis; NC_020317 Magnolia officinalis; NC_023236 Magnolia pyramidata; NC_023240 Magnolia salicifolia; NC_023241 Magnolia sinica; NC_023242 Magnolia sprengeri; NC_024027 Magnolia tripetala; NC_024545 Magnolia yunnanensis; NC_010433 Manihot esculenta; NC_017006 Mankyua chejuensis; NC_030577 Maranthes gabunensis; NC_030578 Maranthes glabra; NC_030579 Maranthes kerstingii; NC_001319 Marchantia polymorpha; NC_022137 Marsilea crenata; NC_025549 Marvania geminata; NC_026541 Masdevallia coccinea; NC_026777 Masdevallia picturata; NC_029947 Mauritia flexuosa; NC_027153 Medicago hybrida; NC_027154 Medicago papillosa; NC_012615 Megaleranthis saniculifolia; NC_030489 Megathyrsus maximus; NC_027477 Melica mutica; NC_027478 Melica subulata; NC_029430 Meliosma aff; NC_029049 Mesembryanthemum crystallinum; NC_024169 Mesotaenium endlicherianum; NC_029214 Metanarthecium luteoviride; NC_022812 Metapanax delavayi; NC_027423 Metasequoia glyptostroboides; NC_029959 Metroxylon warburgii; NC_023239 Michelia odora; NC_012575 Micromonas sp; NC_025537 Microthamnion

kuetzingianum; NC_016708 Millettia pinnata; NC_028720 Miscanthus sacchariflorus; NC_028721 Miscanthus sinensis; NC_025237 Monachather paradoxus; NC_012101 Monomastix sp; NW_014013626 Monoraphidium neglectum; NC_029704 Monotropa hypopitys; NC_029694 Monsonia emarginata; NC_029830 Monsonia marlothii; NC_014582 Monsonia speciosa; NC_028009 Morinda officinalis; NC_008359 Morus indica; NC_025772 Morus mongolica; NC_027110 Morus notabilis; NC_028439 Musa balbisiana; NC_022926 Musa textilis; NC_029671 Mychonastes homosphaera; NC_028579 Mychonastes jurisii; NC_025525 Myrmecia israelensis; NC_023120 Nageia nagi; NC_021936 Najas flexilis; NC_008336 Nandina domestica; NC_009275 Nasturtium officinale; NC_015605 Nelumbo lutea; NC_025339 Nelumbo nucifera; NC_030580 Neocarya macrophylla; NC_029670 Neochloris aquatica; NC_025535 Neocystis brevis; NC_026963 Neohouzeaua sp; NC_026964 Neololeba atra; NC_016471 Neottia nidus; NC_024829 Nephroselmis astigmatica; NC_000927 Nephroselmis olivacea; NC_025656 Nerium oleander; NC_030356 Netrium digitus; NC_024262 Neyraudia reynaudiana; NC_007500 Nicotiana sylvestris; NC_001879 Nicotiana tabacum; NC_007602 Nicotiana tomentosiformis; NC_016068 Nicotiana undulata; NC_020259 Nothoceros aenigmaticus; NC_008788 Nuphar advena; NC_026979 Nyholmiella obtusifolia; NC_006050 Nymphaea alba; NC_024542 Nymphaea mexicana; NC_029958 Nypa fruticans; NC_011031 Oedogonium cardiacum; NC_010358 Oenothera argillicola; NC_010361 Oenothera biennis; NC_010360 Oenothera glazioviana; NC_029211 Oenothera grandiflora; NC_029212 Oenothera oakesiana; NC_010362 Oenothera parviflora; NC_030532 Oenothera villaricae; NC_015401 Olea europaea; NC_015604 Olea europaea; NC_015623 Olea europaea; NC_013707 Olea europaea; NC_015608 Olea woodiana; NC_024722 Oligostachyum shiuyingianum; NC_009267 Olimarabidopsis pumila; NC_026965 Olmeca reflexa; NC_008099 Oltmannsiellopsis viridis; NC_024165 Olyra latifolia; NC_014056 Oncidium Gower; NC_028148 Oncidium sphacelatum; NC_025657 Oncinotis tenuiloba; NC_030490 Oncorachis ramosa; NC_028580 Oogamochlamys gigantea; NC_020147 Ophioglossum californicum; NC_030491 Oplismenus hirtellus; NC_025651 Orobanche californica; NC_024845 Orobanche crenata; NC_023464 Orobanche gracilis; NC_026212 Orthotrichum rogeri; NC_024608 Oryza australiensis; NC_027460 Oryza barthii; NC_030596 Oryza brachyantha; NC_024175 Oryza glaberrima; NC_027461 Oryza glumipatula; NC_027462 Oryza longistaminata; NC_016927 Oryza meridionalis; NC_030298 Oryza minuta; NC_005973 Oryza nivara; NC_027463 Oryza officinalis; NC_027676 Oryza punctata; NC_017835 Oryza rufipogon; NC_001320 Oryza sativa; NC_008155 Oryza sativa; NC_027678 Oryza sativa; NC_027479 Oryzopsis asperifolia; NC_024157 Osmundastrum cinnamomeum; NC_028618 Ostericum grosseserratum; NC_008289 Ostreococcus tauri; NC_028349 Ostrya rehderiana; NC_027960 Osyris alba; NC_030492 Otachyrium versicolor; NC_026971 Otatea acuminata; NC_028631 Otatea glauca; NC_025529 Pabia signiensis; NC_021102 Pachycladon cheesemanii; NC_018565 Pachycladon enysii; NC_029433 Pachysandra terminalis; NC_026076 Paeonia obovata; NC_006290 Panax ginseng; NC_028703 Panax japonicus; NC_026447 Panax notoginseng; NC_027456 Panax quinquefolius; NC_030598 Panax stipuleanatus; NC_028704 Panax vietnamensis; NC_030493 Panicum capillare; NC_029732 Panicum miliaceum; NC_015990 Panicum virgatum; NC_029434 Papaver somniferum; NC_026779 Paphiopedilum armeniacum; NC_026776 Paphiopedilum niveum; NC_012978 Parachlorella kessleri; NC_025540 Paradoxia multiseta; NC_030517 Parastemon urophyllus; NC_027491 Pariana campestris; NC_026972 Pariana radiciflora; NC_024067 Parinari campestris; NC_030581 Parinari capensis; NC_030582 Parinari curatellifolia; NC_030583 Parinari oblongifolia; NC_024560 Paris verticillata; NC_013553 Parthenium argentatum; NC_030494 Paspalidium geminatum; NC_030614 Paspalum dilatatum; NC_030495 Paspalum fimbriatum; NC_030496 Paspalum glaziovii; NC_027450 Pastinaca pimpinellifolia; NC_029700 Pedicularis ishidoyana; NC_016733 Pedinomonas minor; NC_025530 Pedinomonas tuberculata; NC_023261 Pelargonium alternans; NC_028053 Pelargonium australe; NC_028052 Pelargonium cotyledonis; NC_028051 Pelargonium dichondrifolium; NC_008454 Pelargonium x; NC_019628 Pellia endiviifolia; NC_016921 Pentactina rupicola; NC_025658 Pentalinon luteum; NC_023086 Penthorum chinense; NC_023356 Petrosavia stellaris; NC_028587 Phacotus lenticularis; NC_027480 Phaenosperma globosum; NC_007499 Phalaenopsis aphrodite; NC_017609 Phalaenopsis equestris; NC_025593 Phalaenopsis hybrid; NC_027481 Phalaris arundinacea; NC_023245 Pharus lappulaceus; NC_021372 Pharus latifolius;

NC_009259 Phaseolus vulgaris; NC_023132 Phelipanche purpurea; NC_023465 Phelipanche ramosa; NC_027482 Phleum alpinum; NC_013991 Phoenix dactylifera; NC_028149 Phragmipedium longifolium; NC_022958 Phragmites australis; NC_015817 Phyllostachys edulis; NC_015826 Phyllostachys nigra; NC_016699 Phyllostachys propinqua; NC_024669 Phyllostachys sulphurea; NC_029820 Phyllostegia velutina; NC_026570 Physalis peruviana; NC_005087 Physcomitrella patens; NC_029957 Phytelephas aequatorialis; NC_021456 Picea abies; NC_029374 Picea jezoensis; NC_016069 Picea morrisonicola; NC_011152 Picea sitchensis; NC_024828 Picocystis salinarum; NC_029956 Pigafetta elata; NC_029235 Pilostyles aethiopica; NC_029236 Pilostyles hamiltonii; NC_027681 Pinellia ternata; NC_023463 Pinguicula ehlersiae; NC_029847 Pinus armandii; NC_028421 Pinus bungeana; NC_011153 Pinus contorta; NC_011154 Pinus gerardiana; NC_004677 Pinus koraiensis; NC_011155 Pinus krempfii; NC_011156 Pinus lambertiana; NC_011157 Pinus longaeva; NC_021439 Pinus massoniana; NC_011158 Pinus monophylla; NC_011159 Pinus nelsonii; NC_028552 Pinus sibirica; NC_026302 Pinus strobus; NC_028531 Pinus tabuliformis; NC_021440 Pinus taeda; NC_027415 Pinus taiwanensis; NC_001631 Pinus thunbergii; NC_008457 Piper cenocladum; NC_027941 Piper kadsura; NC_027483 Piptochaetium avenaceum; NC_014057 Pisum sativum; NC_030497 Plagiantha tenella; NC_025541 Planctonema lauterbornii; NC_028519 Plantago maritima; NC_028520 Plantago media; NC_008335 Platanus occidentalis; NC_024723 Pleioblastus maculatus; NC_021109 Pleodorina starrii; NC_027484 Poa palustris; NC_023805 Podocarpus lambertii; NC_020361 Podocarpus totara; NC_027276 Podococcus barteri; NC_029881 Pogonatherum paniceum; NC_028429 Polygonatum cyrtonema; NC_029485 Polygonatum sibiricum; NC_028523 Polygonatum verticillatum; NC_008235 Populus alba; NC_024735 Populus balsamifera; NC_024747 Populus euphratica; NC_024734 Populus fremontii; NC_027425 Populus tremula; NC_028504 Populus tremula; NC_009143 Populus trichocarpa; NC_029814 Potamogeton perfoliatus; NC_024817 Prasinoderma coloniale; NC_023833 Praxelis clematidea; NC_026291 Premna microphylla; NC_024543 Primula poissonii; NC_030609 Primula sinensis; NC_021455 Prinsepia utilis; NC_029955 Pritchardia thurstonii; NC_023956 Prunus kansuensis; NC_026981 Prunus maximowiczii; NC_023798 Prunus mume; NC_026982 Prunus padus; NC_014697 Prunus persica; NC_030599 Prunus pseudocerasus; NC_026980 Prunus yedoensis; NC_008114 Pseudendoclonium akinetum; NC_025547 Pseudochloris wilhelmii; NC_030631 Pseudolarix amabilis; NC_029669 Pseudomuriella schumacherensis; NC_020364 Pseudophoenix vinifera; NC_028328 Pseudosasa japonica; NC_016064 Pseudotsuga sinensis; NC_003386 Psilotum nudum; NC_014348 Pteridium aquilinum; NC_015402 Ptilidium pulcherrimum; NC_027485 Puccinellia nuttalliana; NC_023449 Puelia olyriformis; NC_030516 Pugionium cornutum; NC_030515 Pugionium dolabratum; NC_012097 Pycnococcus provasolii; NC_012099 Pyramimonas parkeae; NC_015996 Pyrus pyrifolia; NC_023130 Pyrus spinosa; NC_026790 Quercus aliena; NC_026913 Quercus aquifolioides; NC_029490 Quercus baronii; NC_020152 Quercus rubra; NC_026907 Quercus spinosa; NC_026966 Raddia brasiliensis; NC_008796 Ranunculus macranthus; NC_022927 Ravenala madagascariensis; NC_024827 Retrophyllum piresii; NC_024292 Rhazya stricta; NC_027728 Rheum palmatum; NC_014874 Rhizanthella gardneri; NC_016718 Rhynchoryza subulata; NC_016736 Ricinus communis; NC_027259 Rosmarinus officinalis; NC_030615 Rottboellia cochinchinensis; NC_024168 Roya anglica; NC_030315 Roya obtusa; NC_026444 Sabal domingensis; NC_029431 Sabia yunnanensis; NC_005878 Saccharum hybrid; NC_006084 Saccharum hybrid; NC_029221 Saccharum hybrid; NC_029815 Sagittaria lichuanensis; NC_029954 Salacca ramosiana; NC_027226 Salicornia bigelovii; NC_027224 Salicornia brachiata; NC_027225 Salicornia europaea; NC_028350 Salix babylonica; NC_024681 Salix interior; NC_026462 Salix suchowensis; NC_020431 Salvia miltiorrhiza; NC_025668 Sanionia uncinata; NC_025554 Sapindus mukorossi; NC_026694 Saracha punctata; NC_024713 Sarocalamus faberi; NC_027147 Sartidia dewinteri; NC_027146 Sartidia perrieri; NC_029465 Saussurea involucrata; NC_022813 Schefflera delavayi; NC_029764 Schefflera heptaphylla; NC_029807 Scherffelia dubia; NC_015645 Schizomeris leibleinii; NC_028726 Schrenkiella parvula; NC_023115 Schwalbea americana; NC_029734 Sciadopitys verticillata; NC_027659 Sciaphila densiflora; NC_030282 Scopolia parviflora; NC_026202 Scrophularia takesimensis; NC_027262 Scutellaria baicalensis; NC_028533 Scutellaria insignis; NC_021761 Secale cereale; NC_027837 Sedum oryzifolium; NC_023085 Sedum sarmentosum;

NC_026065 Sedum takesimense; NC_013086 Selaginella moellendorffii; NC_030193 Senna tora; NC_030372 Sequoia sempervirens; NC_029953 Serenoa repens; NC_016433 Sesamum indicum; NC_027451 Seseli montanum; NC_022850 Setaria italica; NC_028075 Setaria viridis; NC_023359 Silene chalcedonica; NC_016729 Silene conica; NC_023358 Silene conoidea; NC_016730 Silene latifolia; NC_016728 Silene noctiflora; NC_023360 Silene paradoxa; NC_016727 Silene vulgaris; NC_028027 Silybum marianum; NC_027732 Sinopodophyllum hexandrum; NC_028209 Sobralia aff; NC_028147 Sobralia callosa; NC_007943 Solanum bulbocastanum; NC_026876 Solanum cheesmaniae; NC_026877 Solanum chilense; NC_028069 Solanum commersonii; NC_026878 Solanum galapagense; NC_026879 Solanum habrochaites; NC_007898 Solanum lycopersicum; AC_000188 Solanum lycopersicum; NC_030207 Solanum melongena; NC_026880 Solanum neorickii; NC_028070 Solanum nigrum; NC_026881 Solanum peruvianum; NC_026882 Solanum pimpinellifolium; NC_008096 Solanum tuberosum; NC_030498 Sorghastrum nutans; NC_008602 Sorghum bicolor; NC_023800 Sorghum timorense; NC_030371 Spathiphyllum kochii; NC_030198 Sphagnum palustre; NC_002202 Spinacia oleracea; NC_015891 Spirodela polyrhiza; NC_030355 Spirogyra maxima; NC_030526 Spondias bahiensis; NC_030527 Spondias tuberosa; NC_029417 Sporobolus heterolepis; NC_027650 Sporobolus maritimus; NC_029416 Sporobolus michauxianus; NC_029825 Stachys byzantina; NC_029822 Stachys chamissonis; NC_029823 Stachys coccinea; NC_029824 Stachys sylvatica; NC_026041 Stangeria eriopus; NC_008116 Staurastrum punctulatum; NC_030499 Steinchisma laxum; NC_029818 Stenogyne bifida; NC_029817 Stenogyne haliakalae; NC_029821 Stenogyne kanehoana; NC_029432 Stephania japonica; NC_025527 Stichococcus bacillaris; NC_008372 Stigeoclonium helveticum; NC_027464 Stipa hymenoides; NC_028444 Stipa lipskyi; NC_029390 Stipa purpurea; NC_022414 Stockwellia quadrifida; NC_030539 Styrax grandiflorus; NC_030260 Swida controversa; NC_029241 Syagrus coronata; NC_012052 Syntrichia ruralis; NC_029952 Tahina spectabilis; NC_016065 Taiwania cryptomerioides; NC_021441 Taiwania flousiana; NC_028738 Takakia lepidozioides; NC_030195 Talipariti hamabo; NC_027955 Tanaecium tetragonolobum; NC_020321 Taxus mairei; NC_020098 Tectona grandis; NC_029897 Tenaxia guillarmodiae; NC_021425 Tetracentron sinense; NC_024291 Tetraphis pellucida; NC_029339 Tetrastigma hemsleyanum; NC_026103 Thalictrum coreanum; NC_024724 Thamnocalamus spathiflorus; NC_014676 Theobroma cacao; NC_030616 Thyridolepis xerophila; NC_025238 Thysanolaena latifolia; NC_028588 Tilia amurensis; NC_028589 Tilia mandshurica; NC_028590 Tilia oliveri; NC_028591 Tilia paucicostata; NC_029813 Tofieldia thibetica; NC_029398 Torreya fargesii; NC_027486 Torreyochloa pallida; NC_010442 Trachelium caeruleum; NC_018569 Trebouxiophyceae sp; NC_028578 Treubaria triappendiculata; NC_024035 Trifolium aureum; NC_025743 Trifolium boissieri; NC_025744 Trifolium glanduliferum; NC_024034 Trifolium grandiflorum; NC_024166 Trifolium meduseum; NC_024036 Trifolium repens; NC_025745 Trifolium strictum; NC_011828 Trifolium subterraneum; NC_023959 Trigonobalanus doichangensis; NC_027185 Trillium cuneatum; NC_027282 Trillium decumbens; NC_027738 Trillium maculatum; NC_027739 Trillium tschonoskii; NC_027487 Trisetum cernuum; NC_029951 Trithrinax brasiliensis; NC_020372 Trithuria inconspicua; NC_002762 Triticum aestivum; NC_025955 Triticum macha; NC_021760 Triticum monococcum; NC_024764 Triticum timopheevii; NC_024814 Triticum turgidum; NC_021762 Triticum urartu; NC_021426 Trochodendron aralioides; NC_030630 Tsuga chinensis; NC_026796 Tydemania expeditionis; NC_013823 Typha latifolia; NC_029040 Ulva fasciata; NC_030312 Ulva linza; NC_030067 Urochloa brizantha; NC_030066 Urochloa decumbens; NC_030069 Urochloa humidicola; NC_030617 Urochloa reptans; NC_030068 Urochloa ruziziensis; NC_021449 Utricularia gibba; NC_025653 Utricularia macrorhiza; NC_029719 Utricularia reniformis; NC_019616 Vaccinium macrocarpon; NC_026778 Vanilla planifolia; NC_029950 Veitchia arecina; NC_022715 Veratrum patulum; NC_030220 Verdigellas peltata; NC_027155 Vicia sativa; NC_021091 Vigna angularis; NC_013843 Vigna radiata; NC_018051 Vigna unguiculata; NC_026986 Viola seoulensis; NC_028012 Viscum album; NC_027959 Viscum crassulae; NC_027829 Viscum minimum; NC_029454 Vitis aestivalis; NC_023790 Vitis rotundifolia; NC_007957 Vitis vinifera; NC_029949 Wallichia densiflora; NC_029974 Washingtonia robusta; NC_025526 Watanabea reniformis; NC_010654 Welwitschia mirabilis; NC_030618 Whiteochloa capillipes; NC_027677 Wisteria floribunda; NC_029406 Wisteria sinensis; NC_015899 Wolffia australiana; NC_015894 Wolffiella lingulata; NC_027235 Wollemia nobilis;

NC_028543 Woodwardia unigemmata; NC_027158 Xerophyllum tenax; NC_025534 Xylochloris irregularis; NC_024725 Yushania levigata; NC_026040 Zamia furfuracea; NC_027939 Zanthoxylum piperitum; NC_030377 Zea diploperennis; NC_030301 Zea luxurians; NC_001666 Zea mays; NC_030300 Zea perennis; NC_030500 Zeugites pittieri; NC_020363 Zingiber spectabile; NC_026967 Zizania aquatica; NC_029401 Zizania latifolia; NC_030299 Ziziphus jujuba; NC_029418 Zoysia macrantha; NC_008117 Zygnema circumcarinatum

Figure S1. A log-log scale graph showing the mitocondrial genome size (in kb) versus (a) total length of MTPTs in kb, (b) total number of MTPTs, or (c) MTPT mtDNA % coverage. Outliers are labeled on the plots. Supporting data is shown in supplementary Table S1. Results from the Spearman non-parametric test are show within each plot.

a.

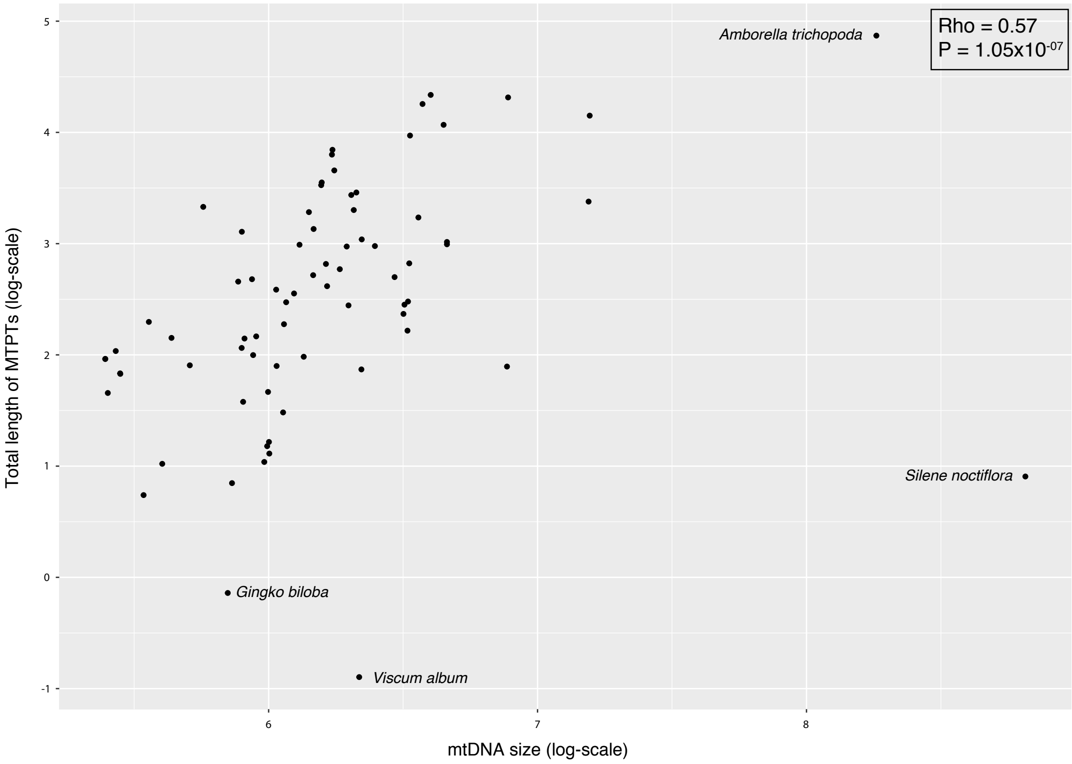

b.

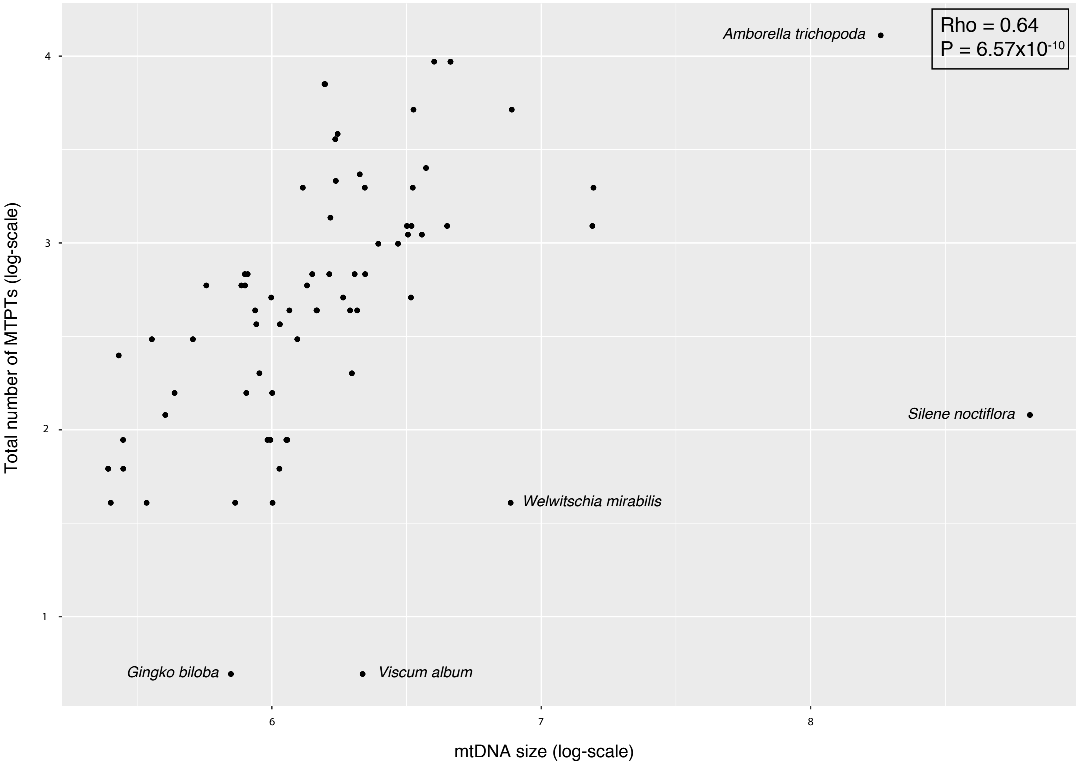

C.

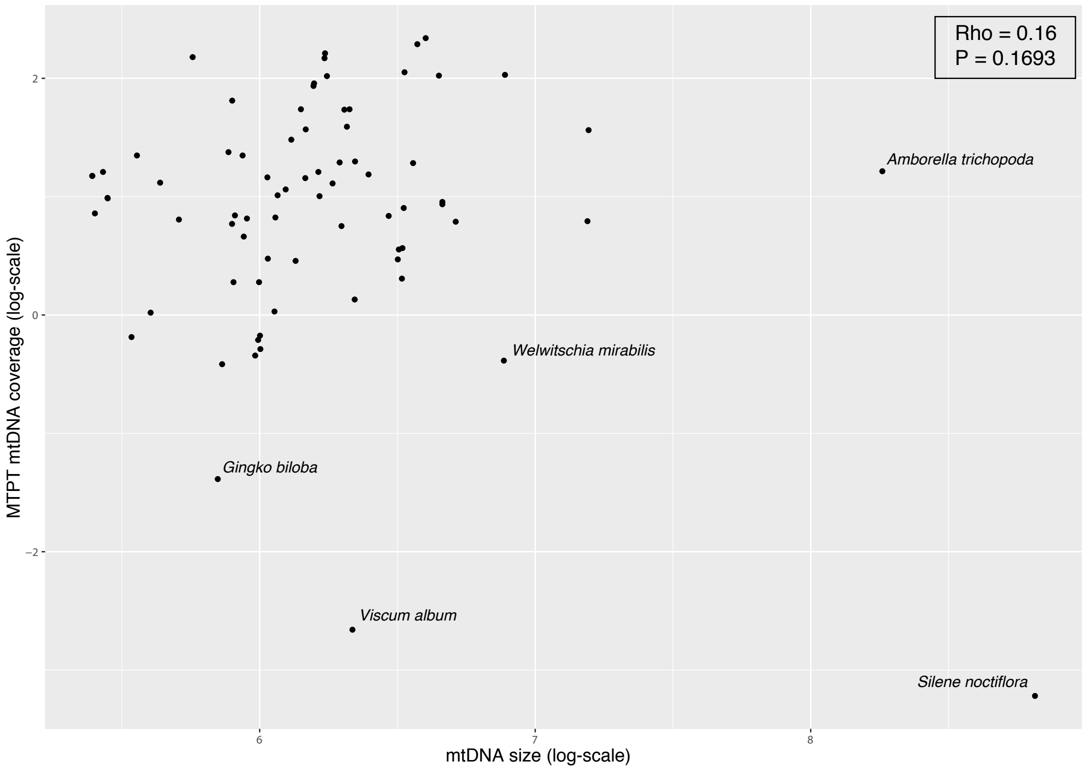
Rho = 0.16
P = 0.1693
Amborella trichopoda
Welwitschia mirabilis
Gingko biloba
Viscum album
Silene noctiflora
MTPT mtDNA coverage (log-scale)
mtDNA size (log-scale)
2
0
−2
6
7
8

Figure S2. Best trees of foreign plastid sequences in mitochondrial genomes of angiosperms. Trees were constructed using RAxML v.8.0.0 under the GTRGAMMA model. Each tree was rooted with *Amborella trichopoda*, if available. Bootstrap support values (1,000 replicates) >50% are shown above the branches. MTPTs and plastid-encoded sequences of the recipient, or the closest species to the recipient, are shown in black and grey bold face, respectively.

a. *Asclepias syriaca* (1) *ndhB-1* (340 nt); (2) *ndhB-1-rps7-rps12* (2,083 nt); (3) non-coding region between *rps7* and *trnV-GAC* (1,281 nt)

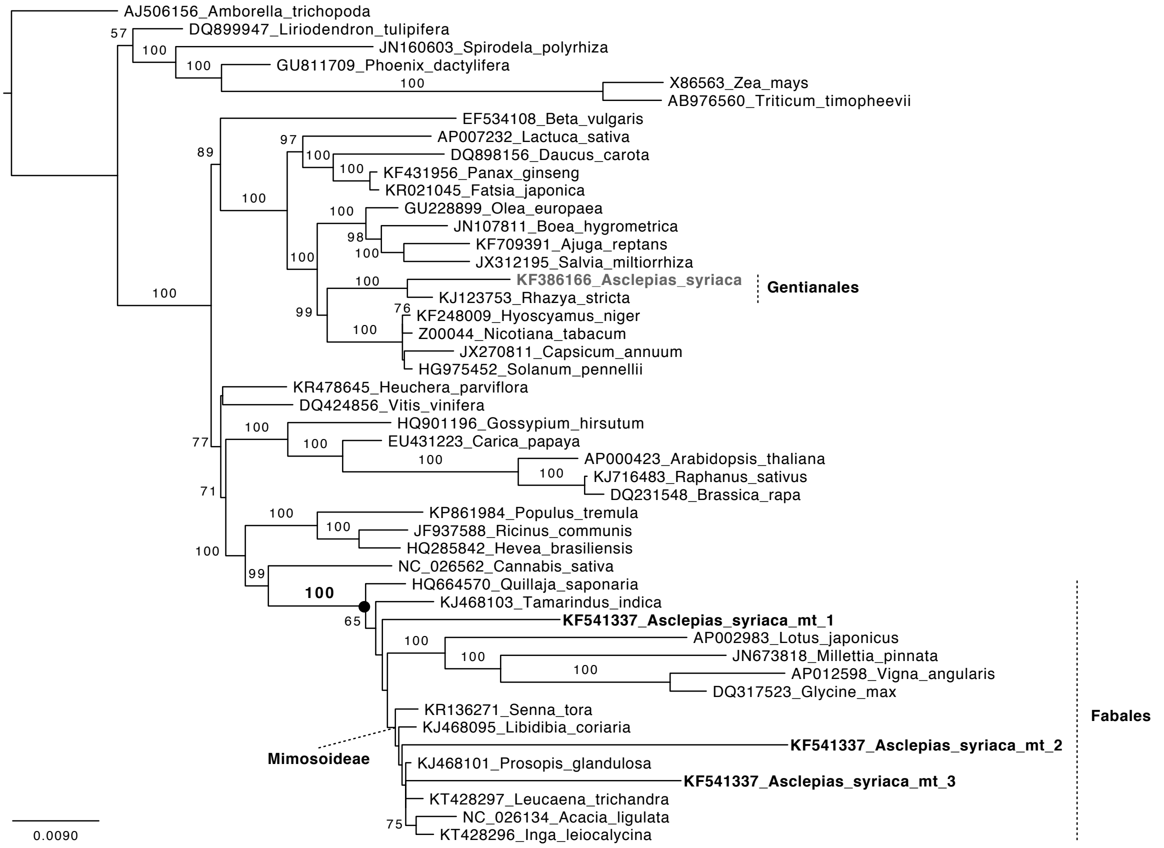

b. *Asclepias syriaca*, *ndhD* (278 nt)

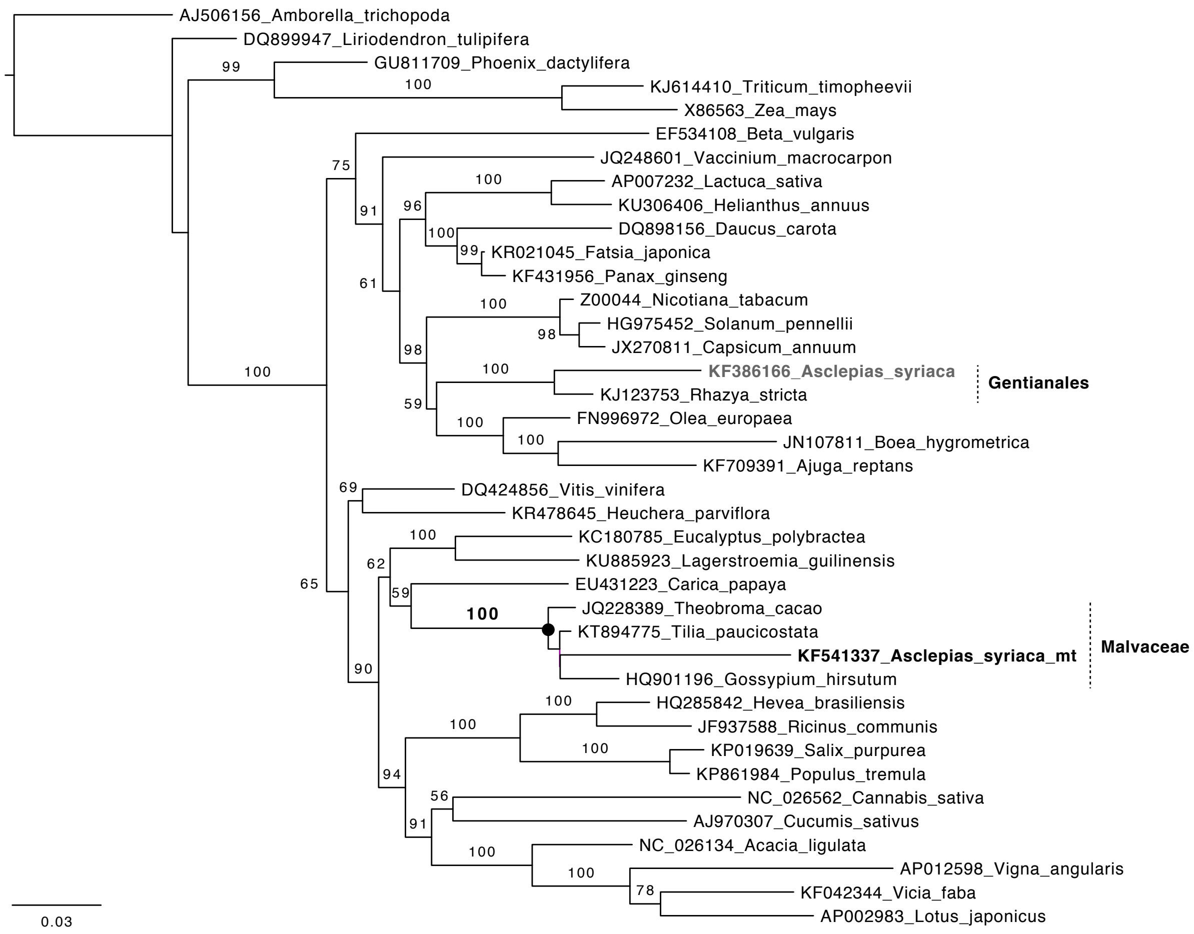

c. *Cucurbita pepo*, *cemA-petA* (1,025 nt)

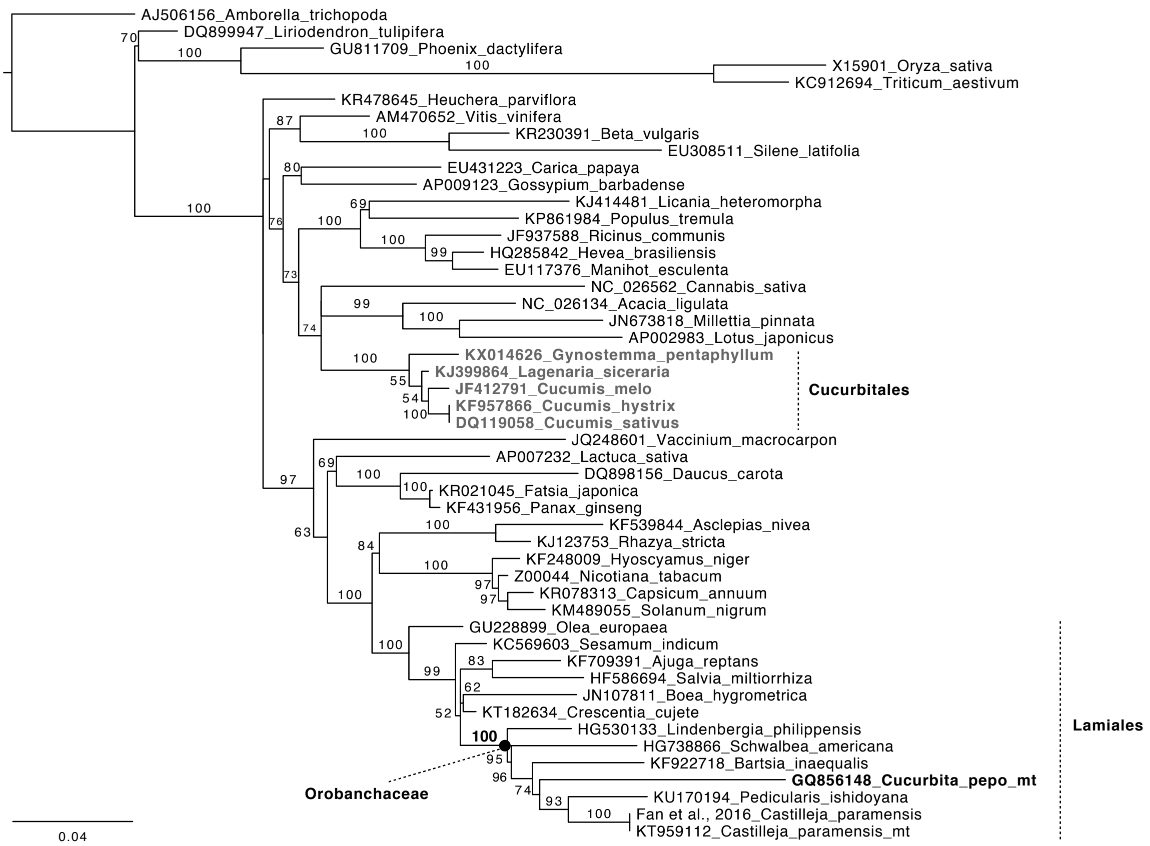

d. *Cucurbita pepo*, *rps7* (1,332 nt)

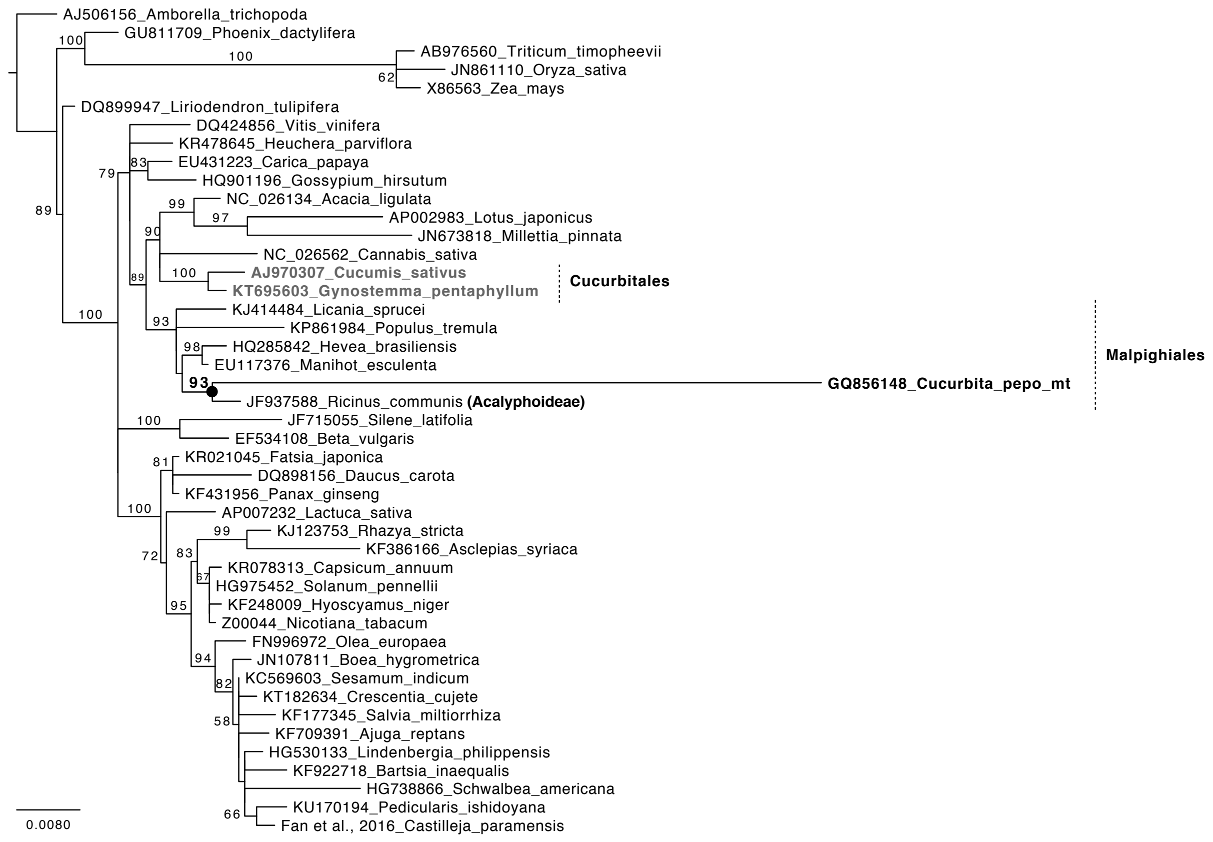

e. *Erythranthe guttata (Mimulus guttatus)*, *rps7* (348 nt)

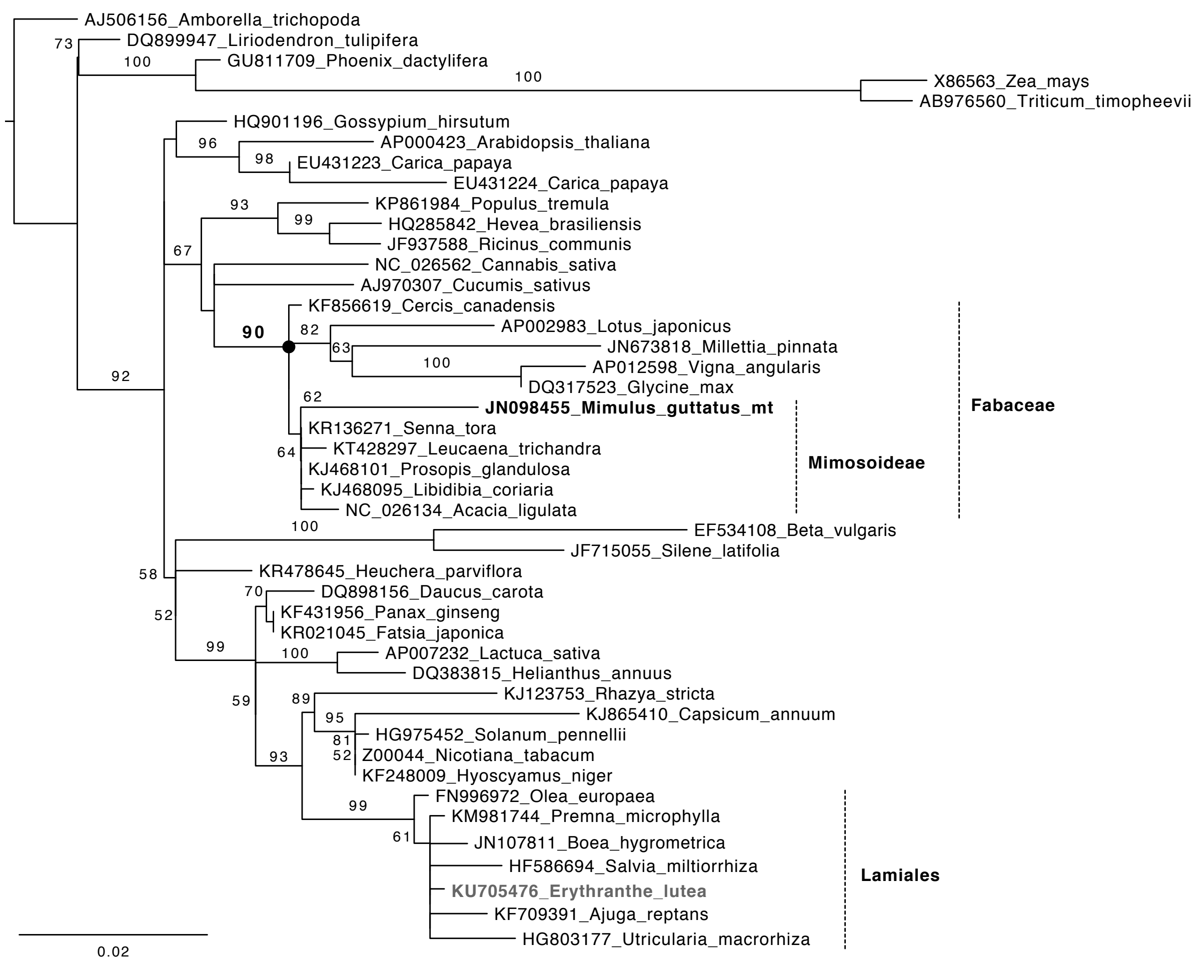

f. *Geranium maderense*, *trnA-Gly, trnG-trnR* (376 nt)

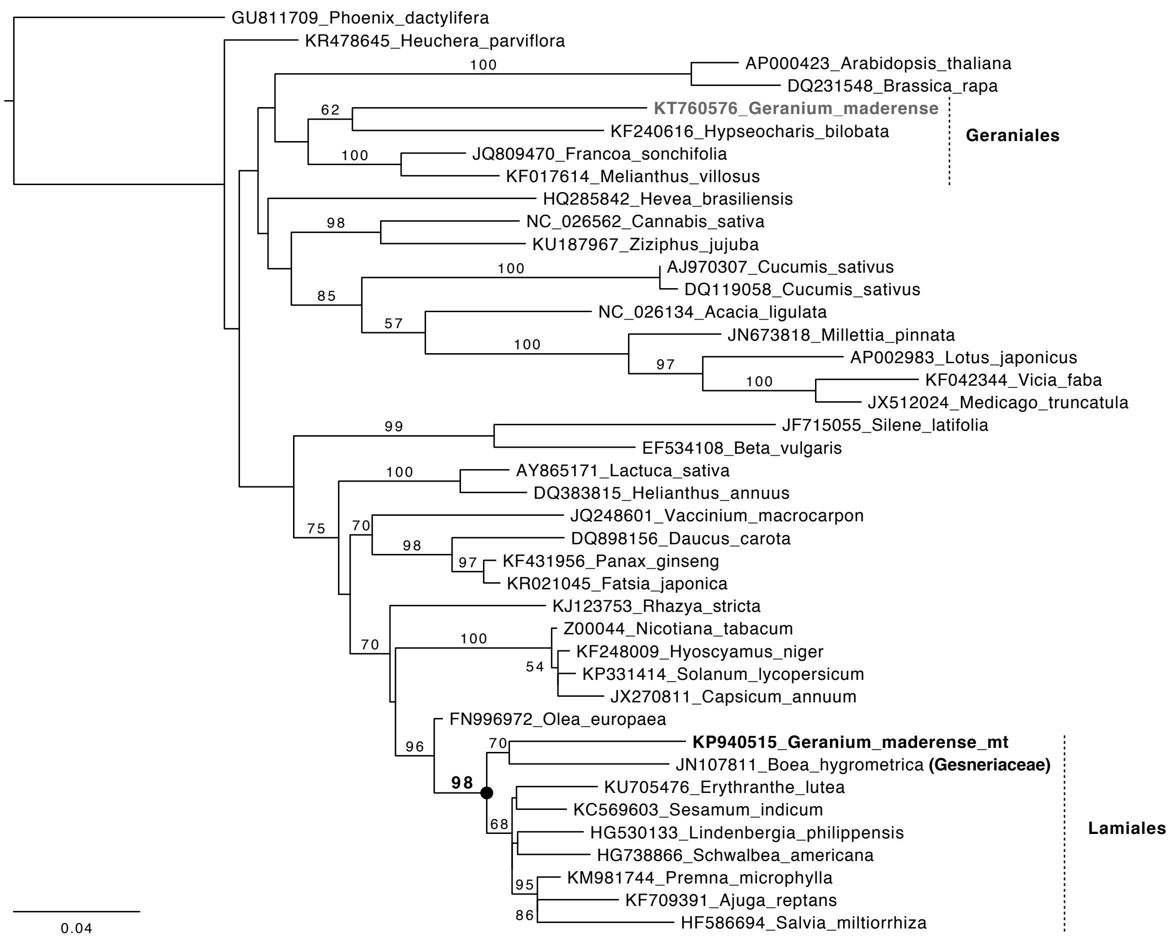

g. *Gossypium hirsutum*, *Gossypium harknessii*, *psbD* (223 nt)

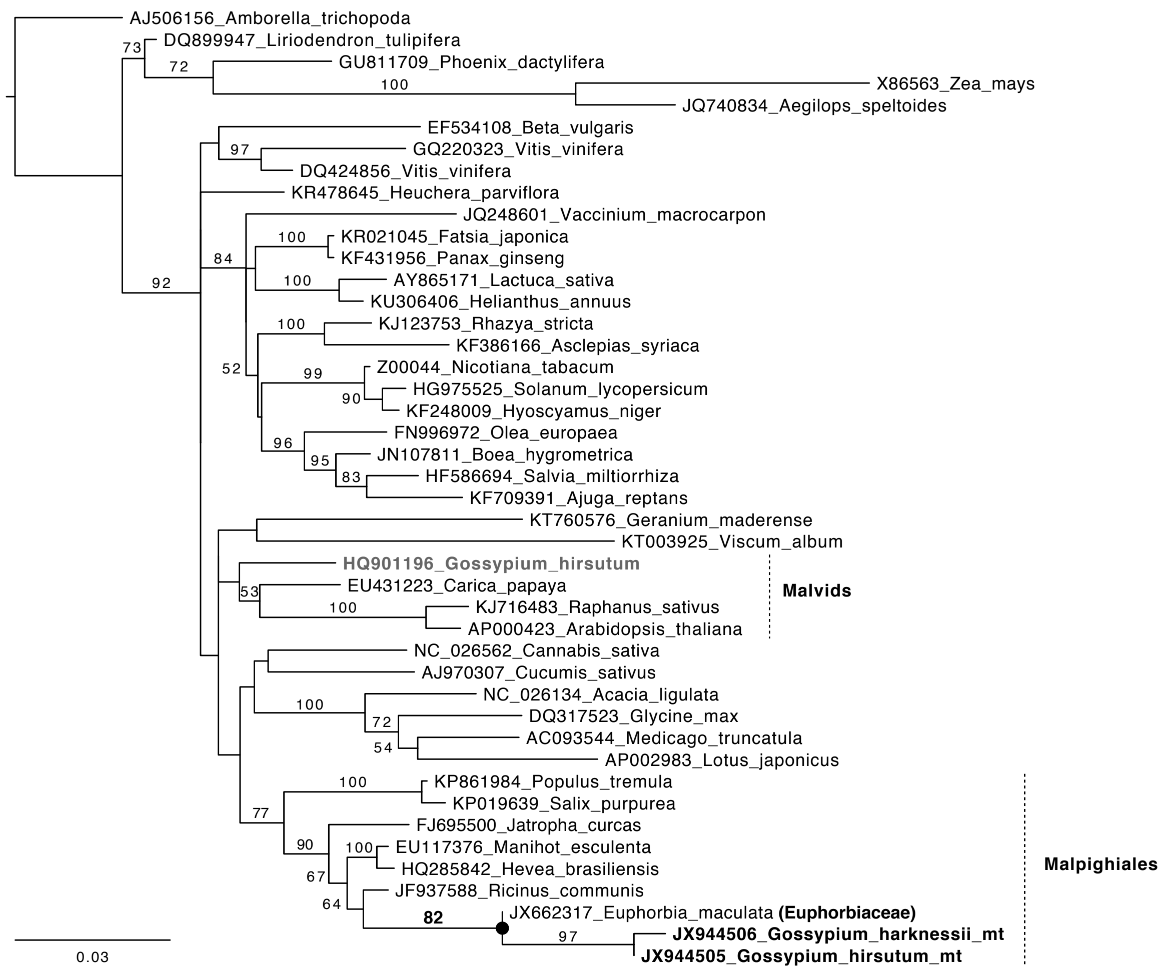

h. *Helianthus annuus*, *infA-rps8*; *rps11* (646 nt)

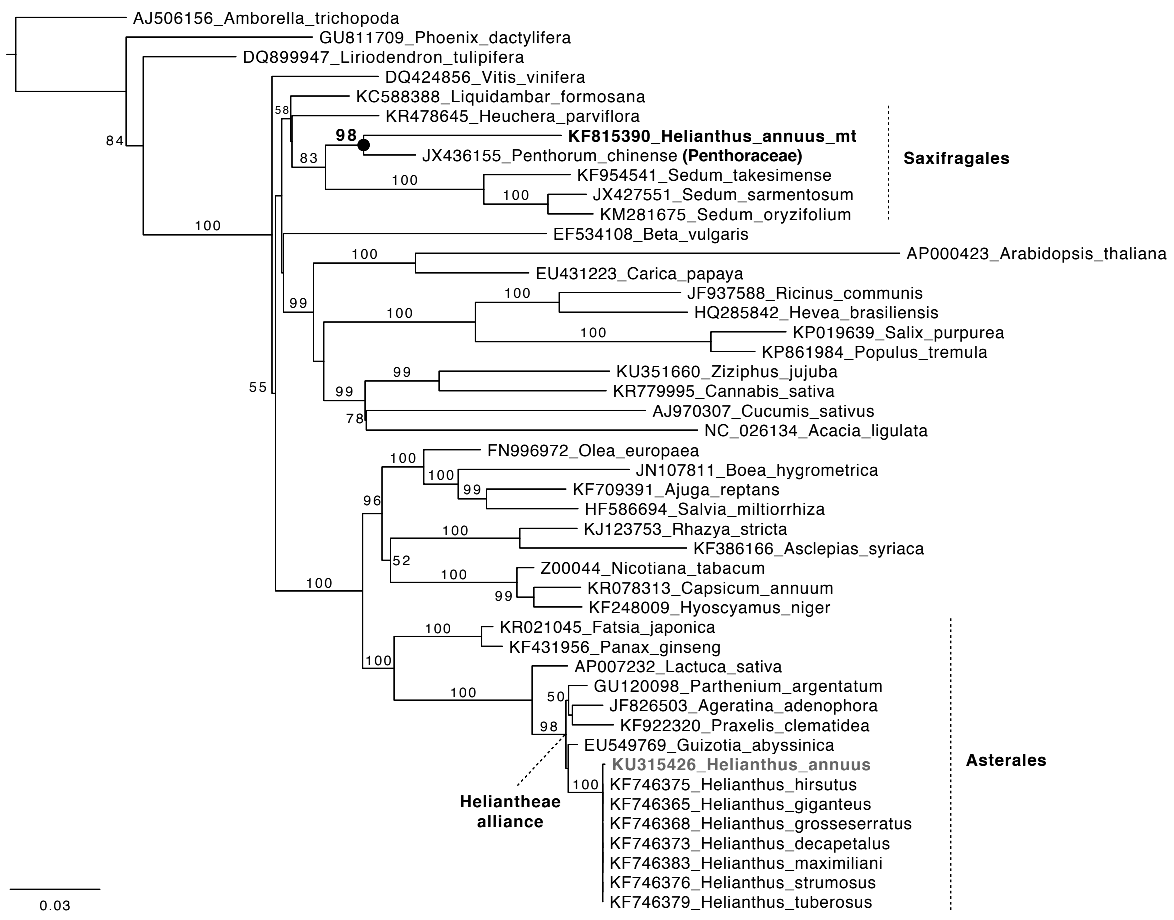

i. *Hyoscyamus niger*, *rps12* (1,974 nt)

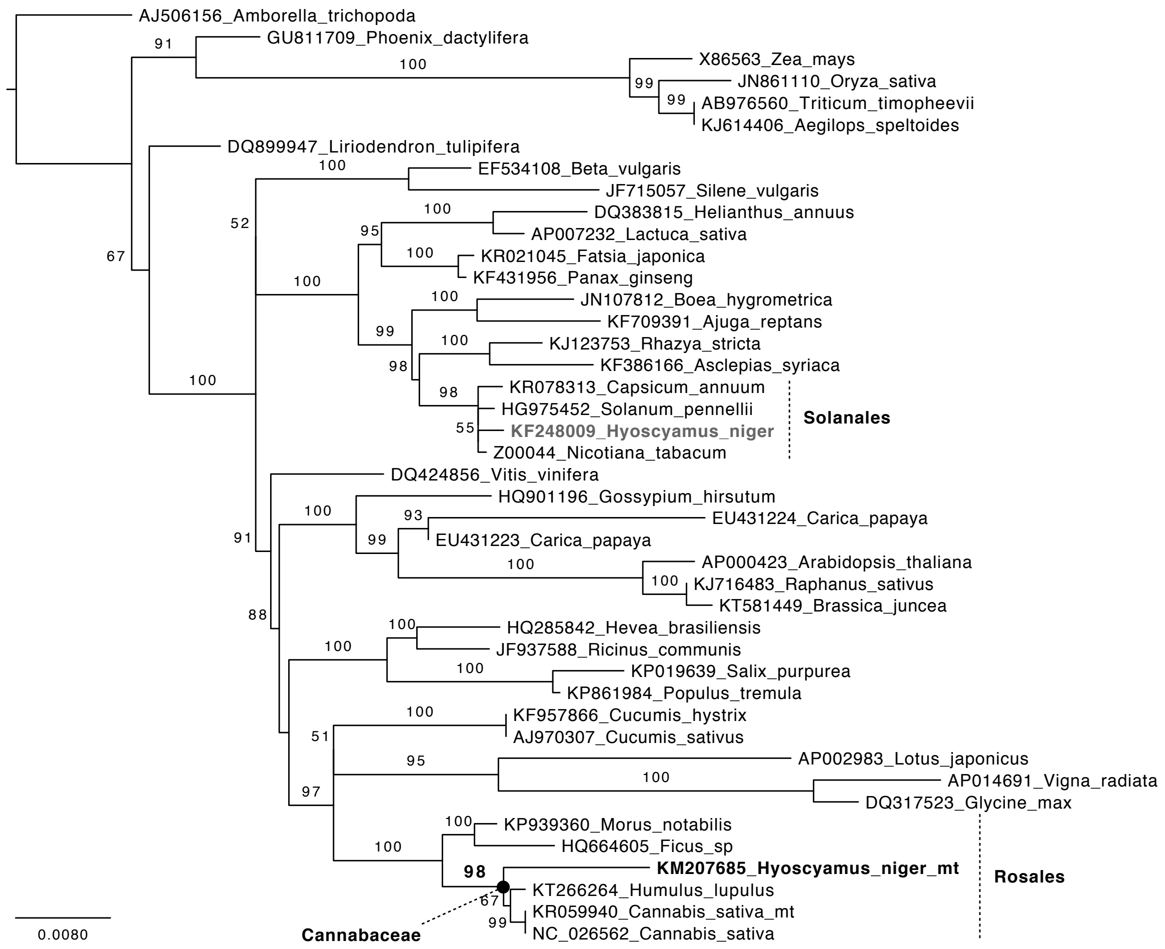

j. *Hyoscyamus niger*, plastid non-coding region (194 nt)

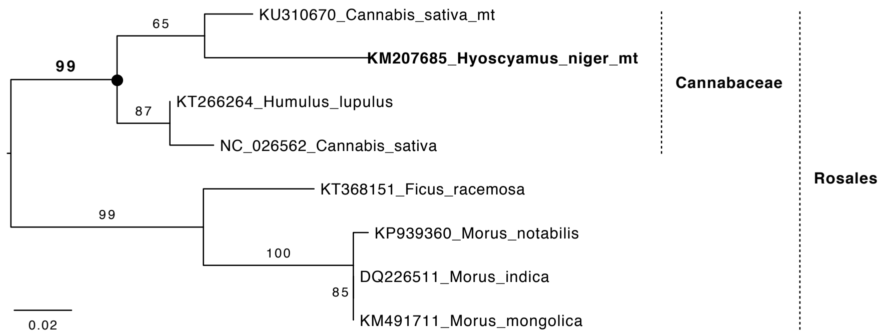

k. *Hyoscyamus niger*, *petB* (1,263 nt)

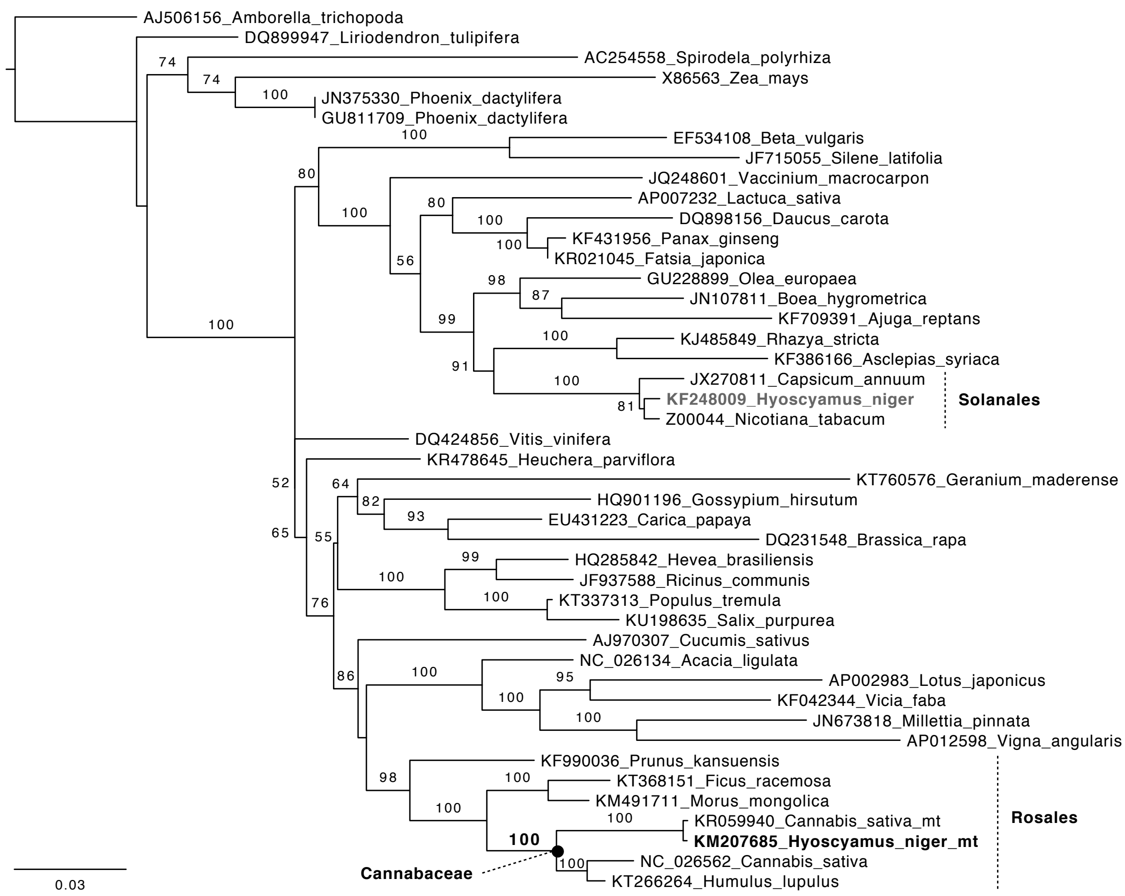

I. *Lophophytum mirabile, rpl2* (fragment_1 245 nt; fragment_2 771 nt)

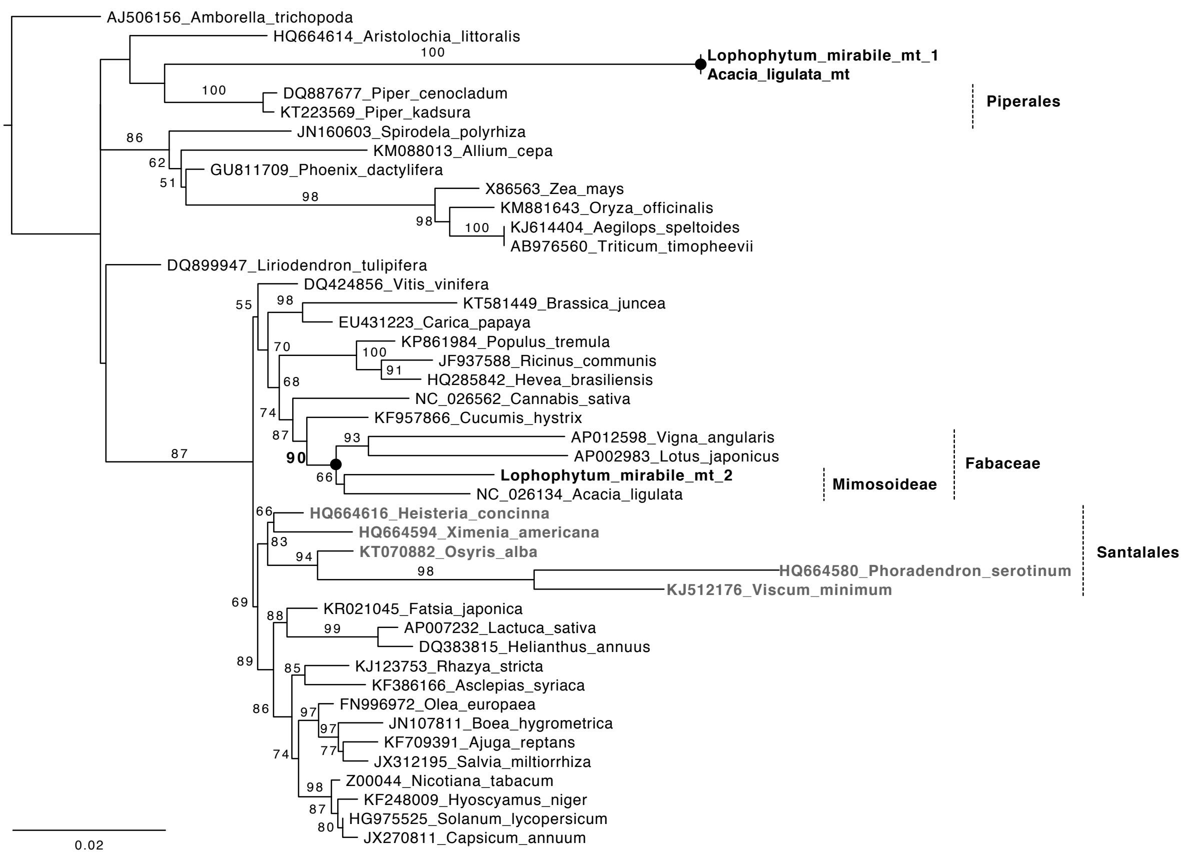

m. *Lophophytum mirabile, rrn23* (726 nt)

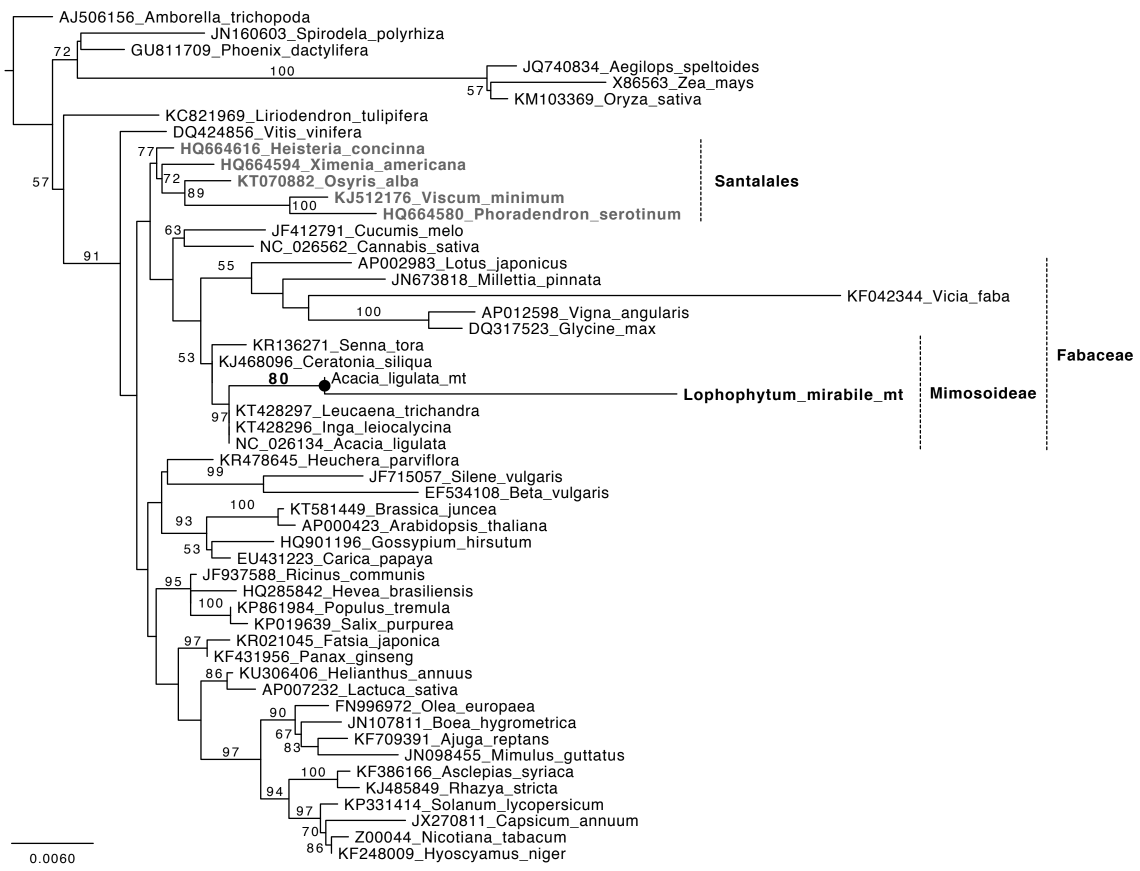

n. *Lophophytum mirabile, psbA* (638 nt)

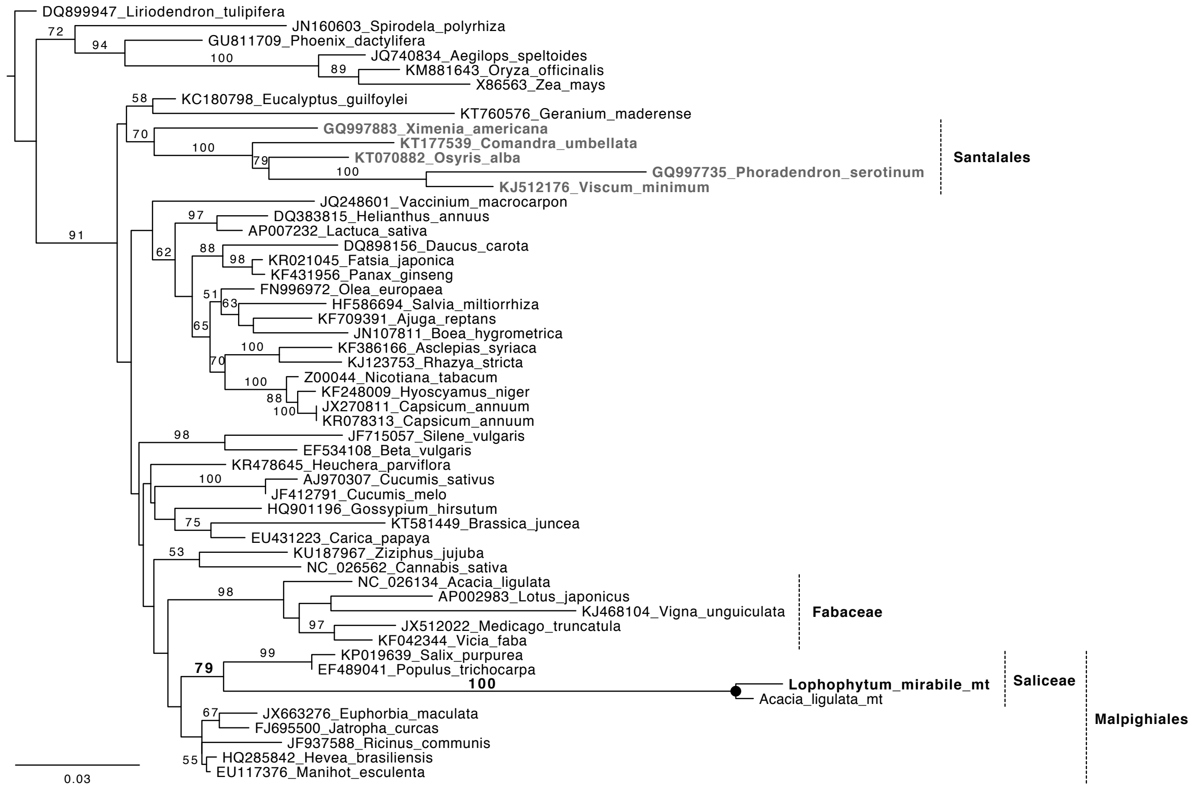

o. *Lophophytum mirabile, rpl16* (520 nt)

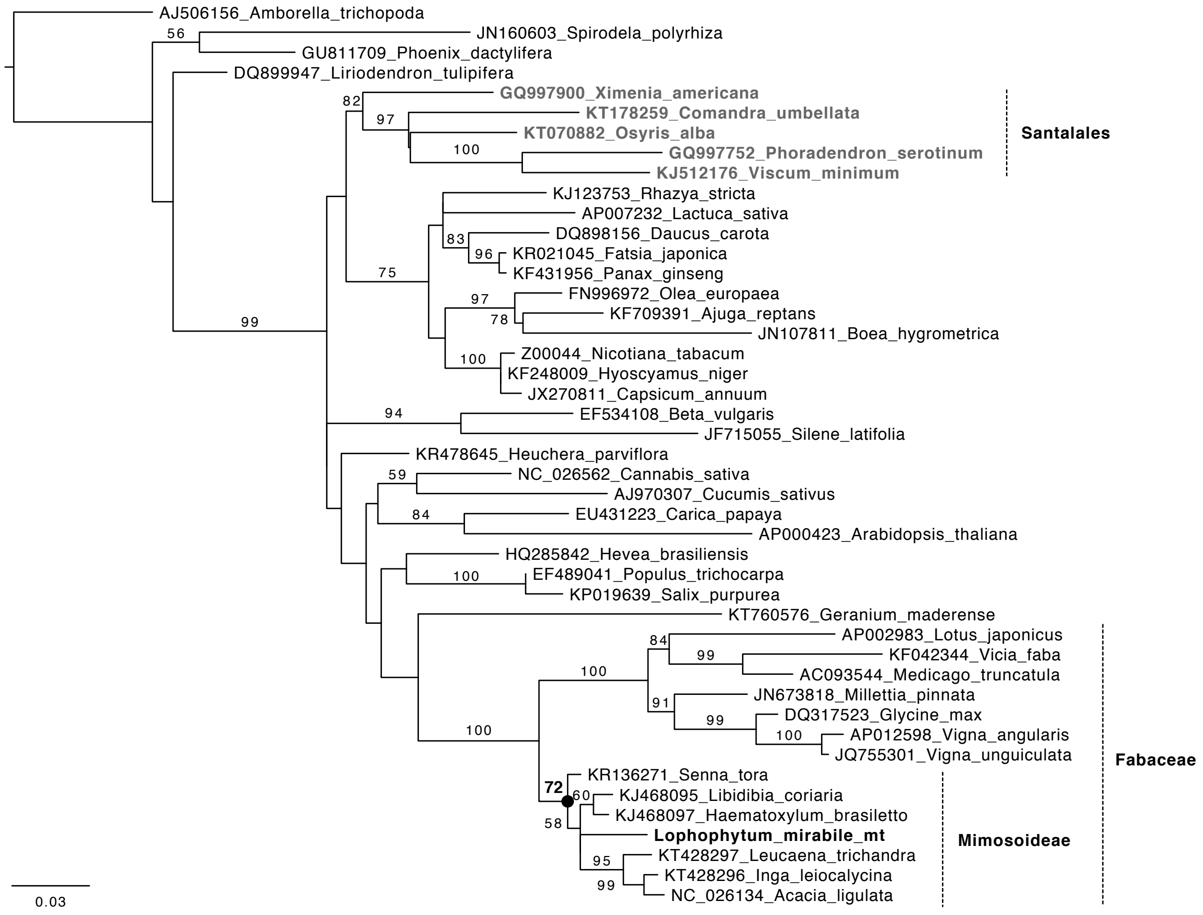

p. *Lophophytum mirabile, petG-trnW* (673 nt)

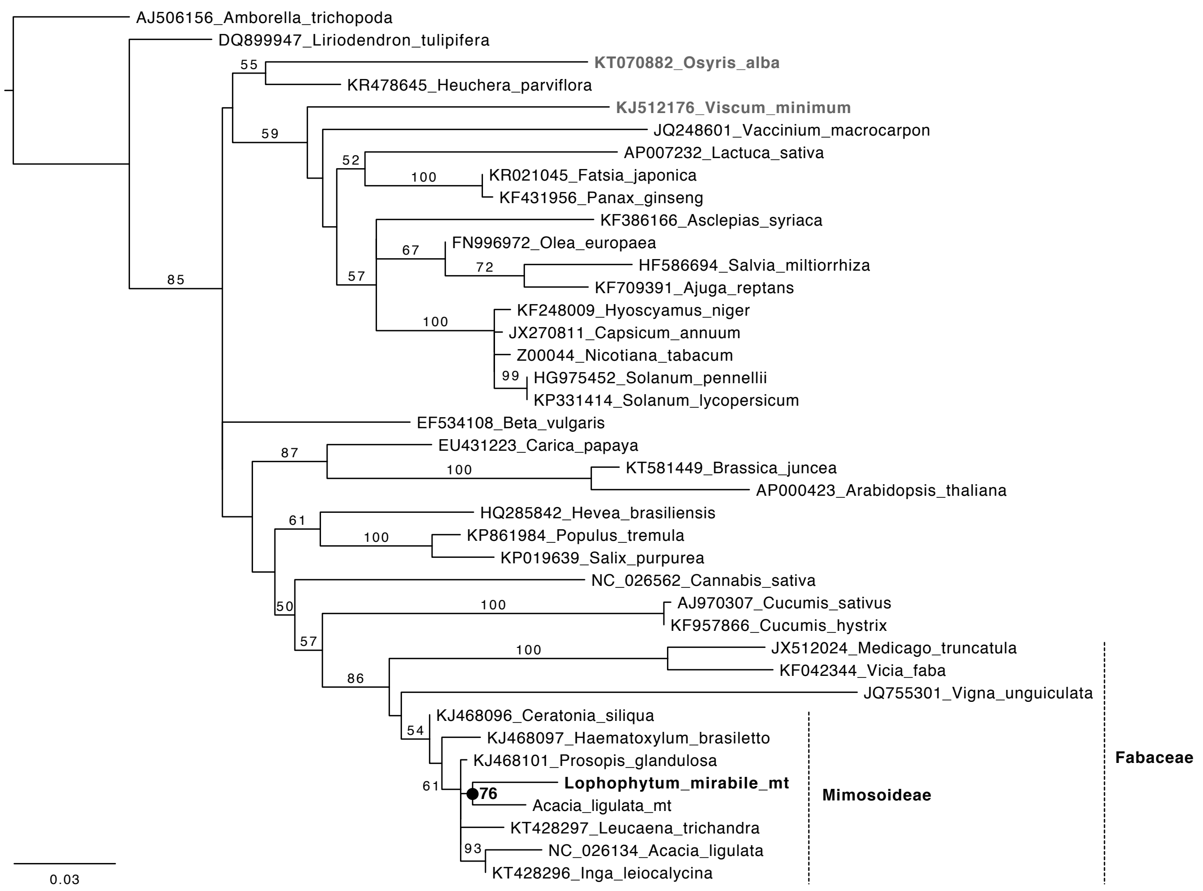

q. *Lophophytum mirabile, rbcL* (269 nt)

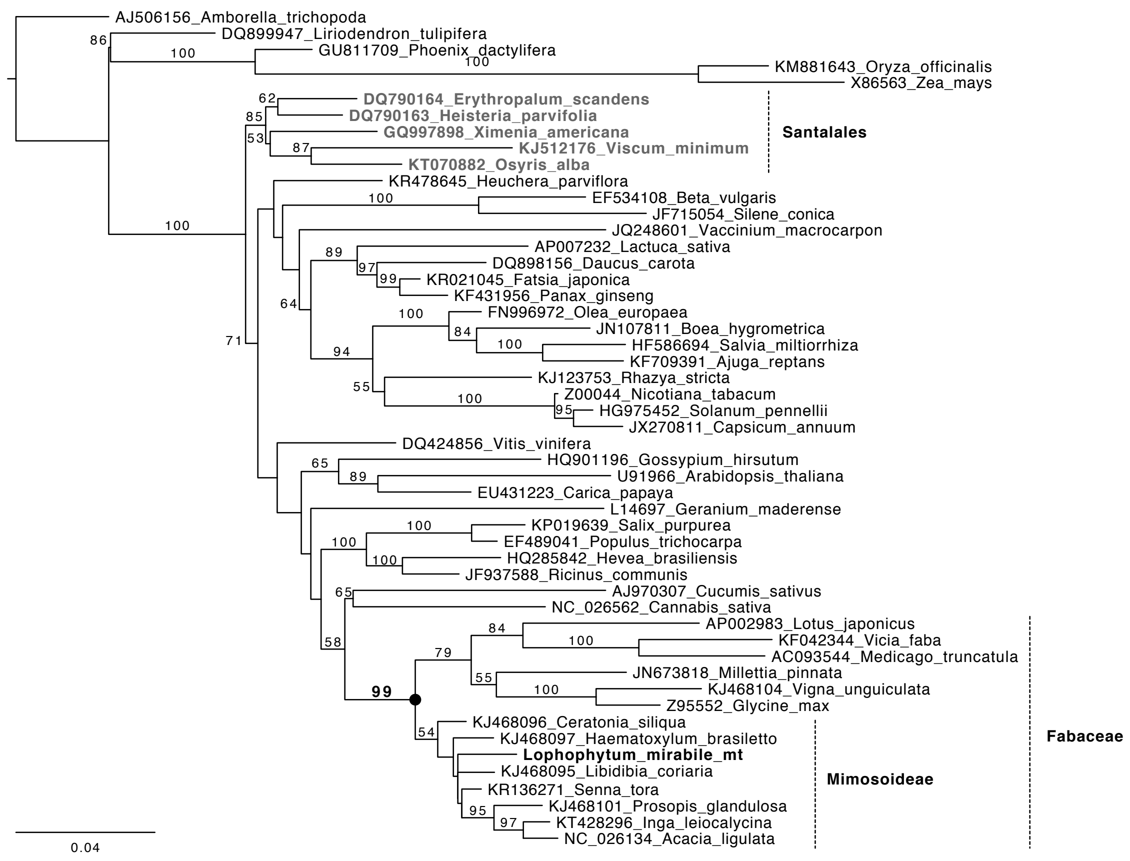

r. *Rhazya stricta, trnI - ycf2* (1,623 nt)

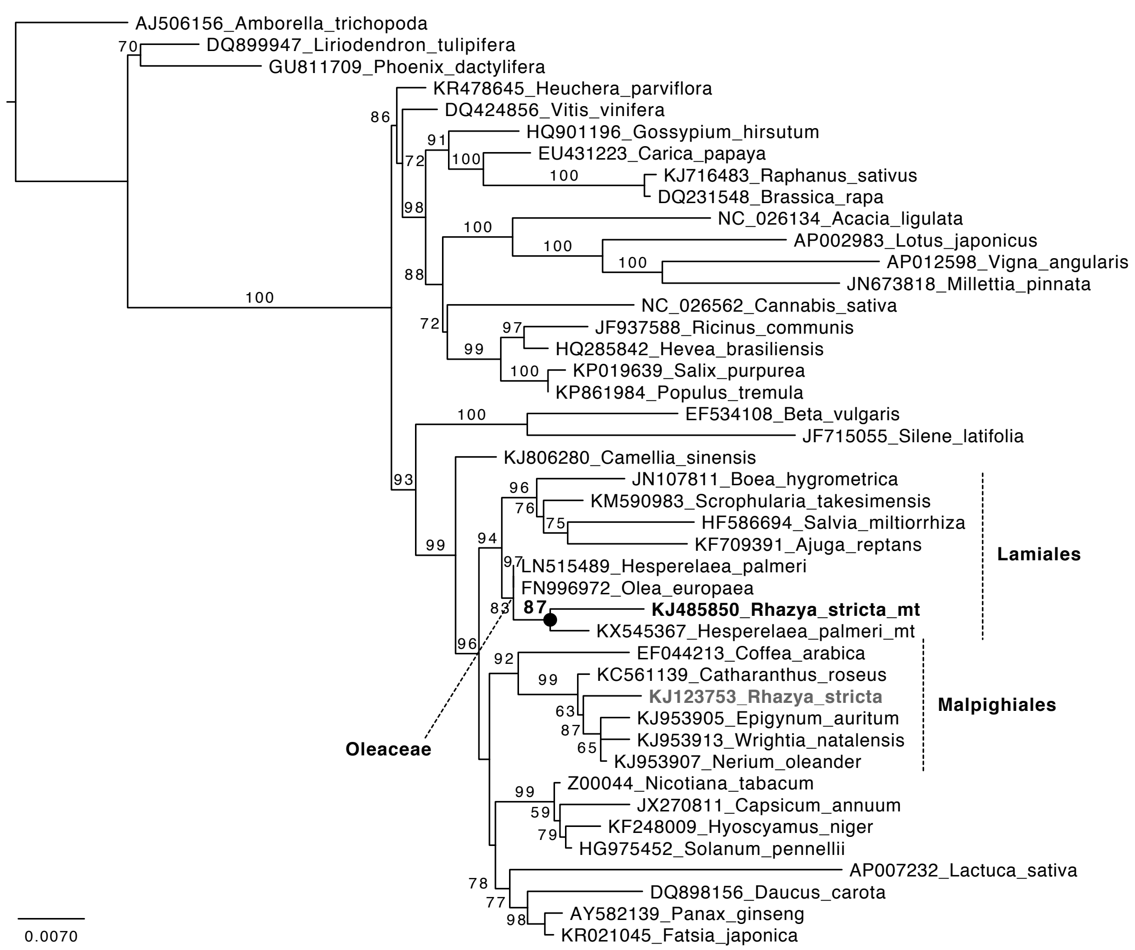

s. *Salvia miltiorrhiza*, *psbA* (350 nt)

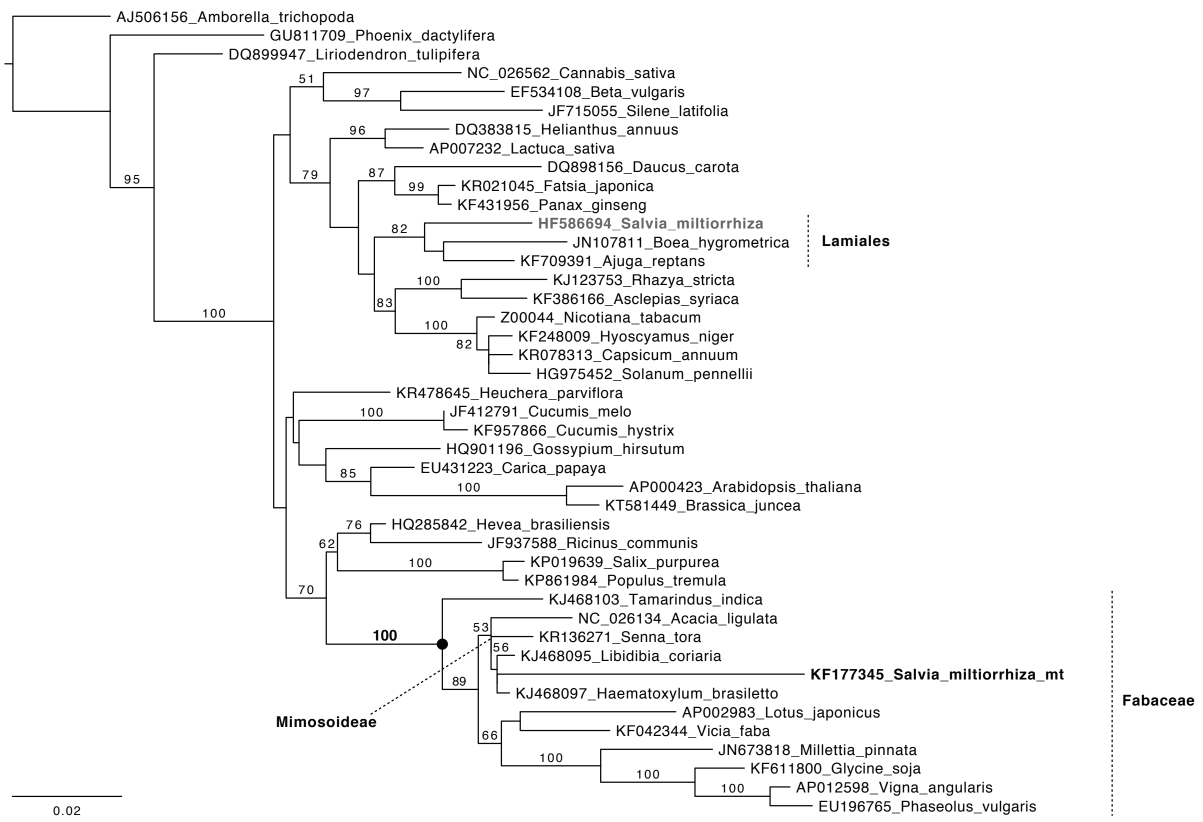

Figure S3. Best trees of regions flanking foreign plastid-derived sequences in mitochondrial genomes of angiosperms. Trees were constructed using RAxML v.8.0.0 under the GTRGAMMA model. Each tree was rooted with *Amborella trichopoda*, if available. Bootstrap support values (1,000 replicates) >50% are shown above the branches. MTPTs and plastid-encoded sequences of the recipient, or the closest species to the recipient, are shown in black and grey bold face, respectively.

a. *Amborella trichopoda*, *rbcL,* 5' flanking region, non-coding region (314 nt)

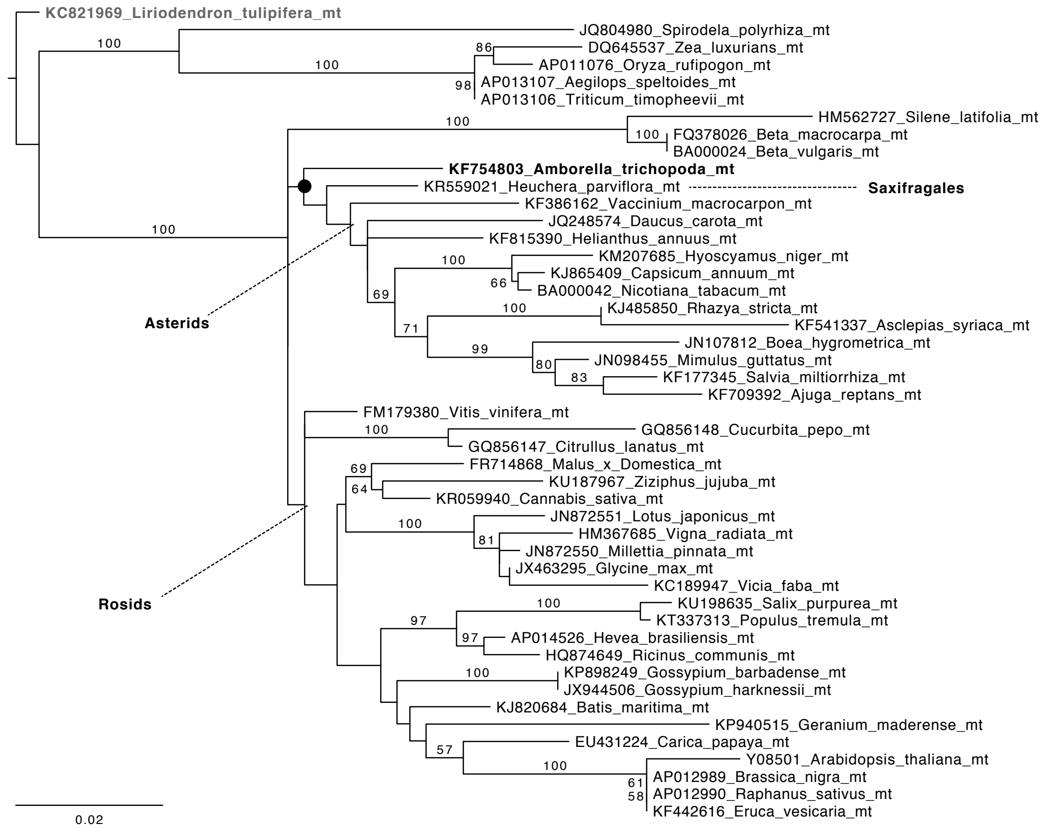

b. *Amborella trichopoda*, *psaA,* 3' flanking region, non-coding region (883 nt)

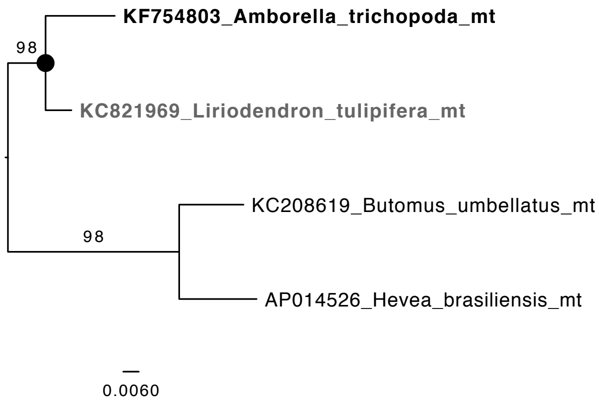

c. *Amborella trichopoda*, *rps7-rps12-trnV-rrnS,* 5’ flanking region, non-coding region (2,185 nt)

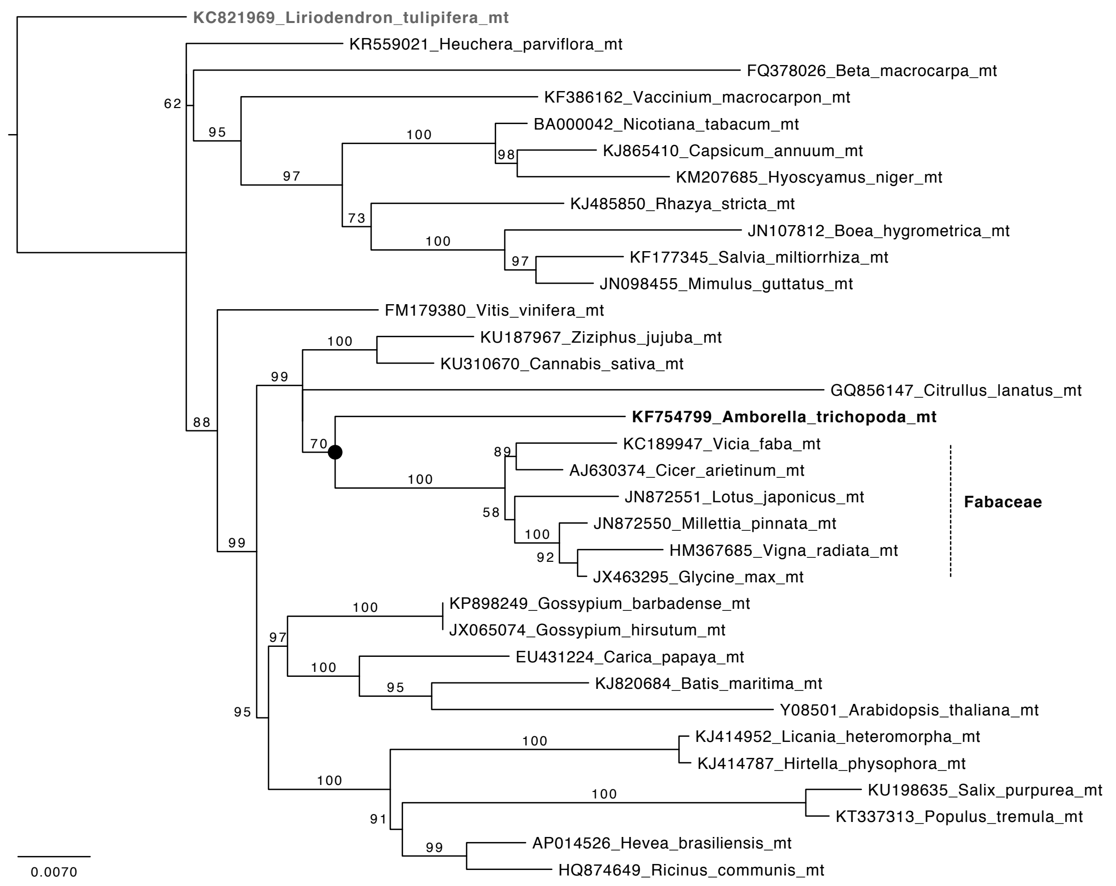

d. *Asclepias syriaca* (fragment 3, non-coding region), 3’ flanking region, non-coding region (196 nt)

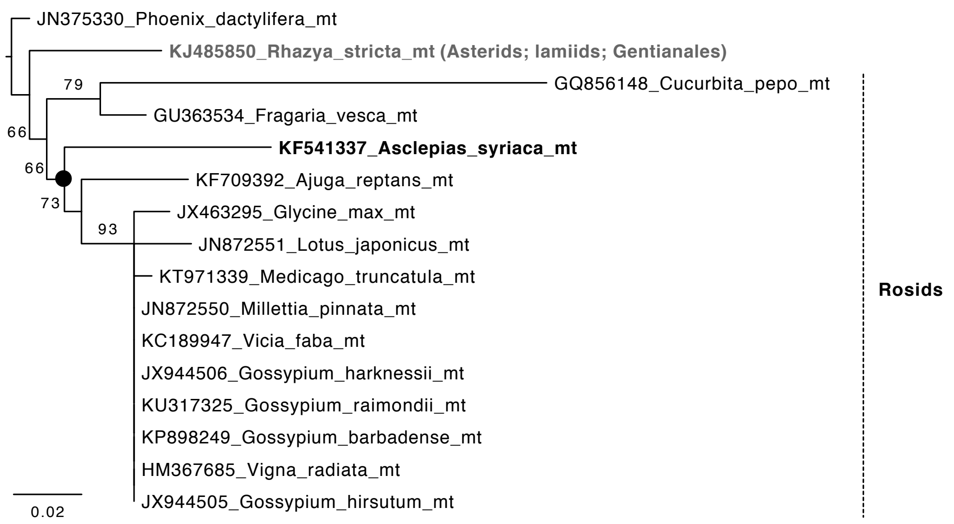

e. *Geranium brycei*, *psaA,* 3’ flanking region, non-coding region (147 nt)

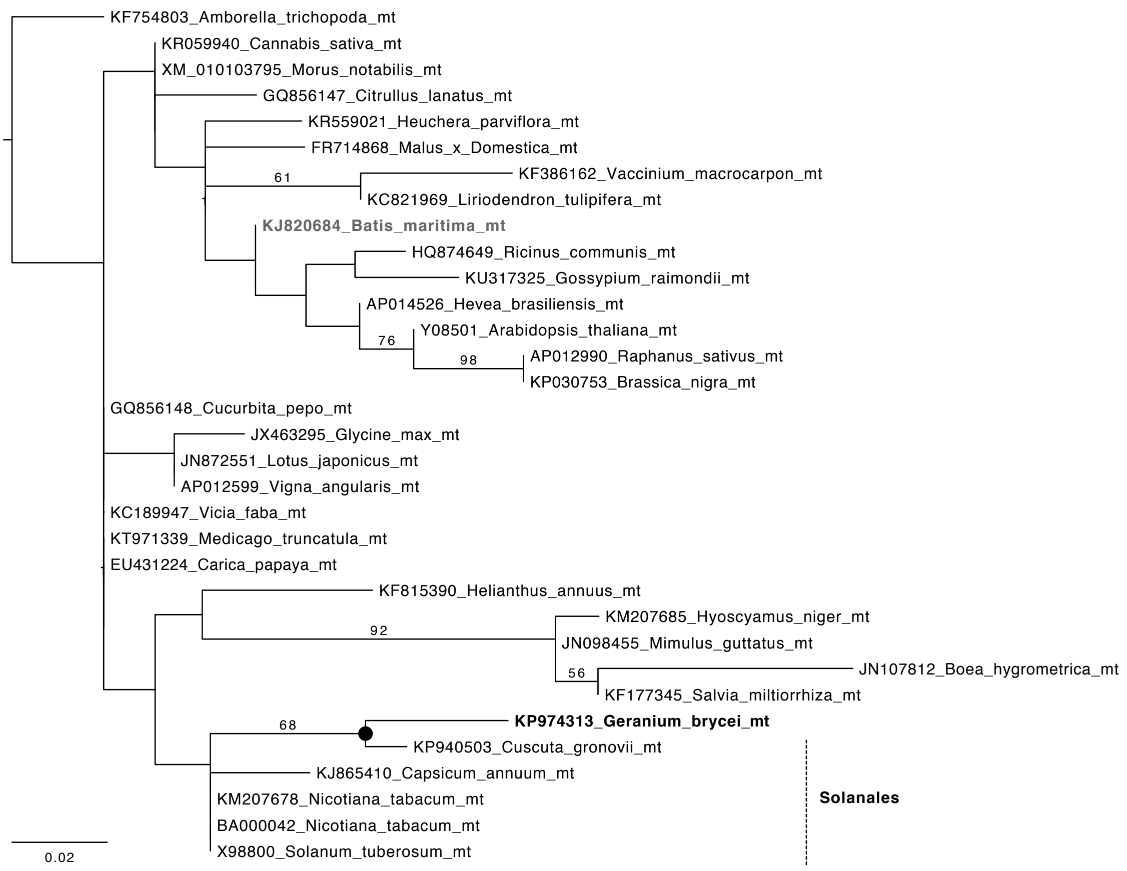

f. *Geranium brycei*, *psaB-rps14,* 3’ flanking region, *trnE* and *trnQ* (825 nt)

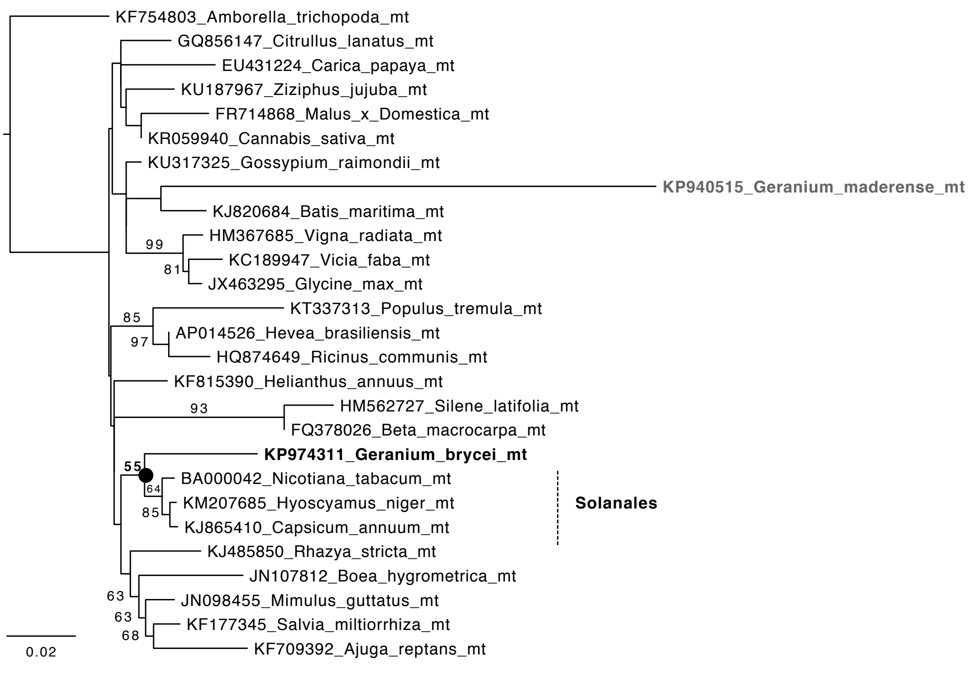

g. *Helianthus annuus, infA-rps8*; *rps11,* 3’ flanking region, non-coding region (240 nt)

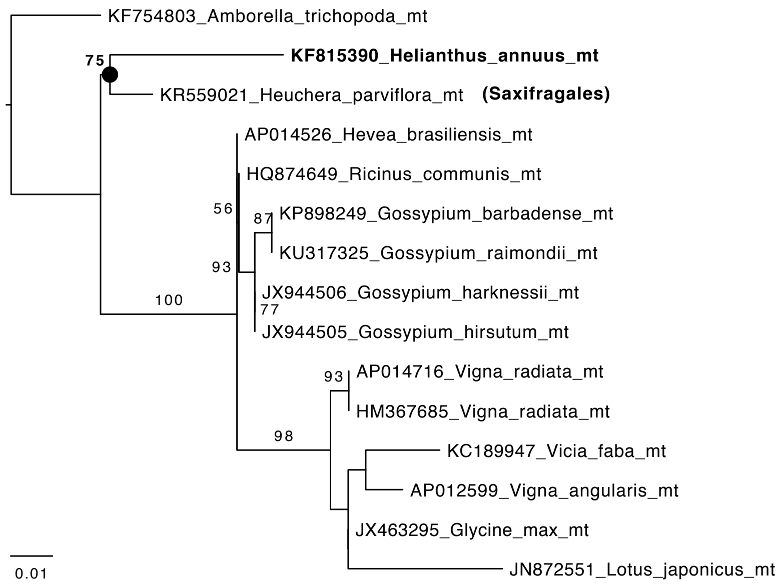

h. *Helianthus annuus*, *infA-rps8*; *rps11,* 5’ flanking region, *coxI* (1,695 nt)

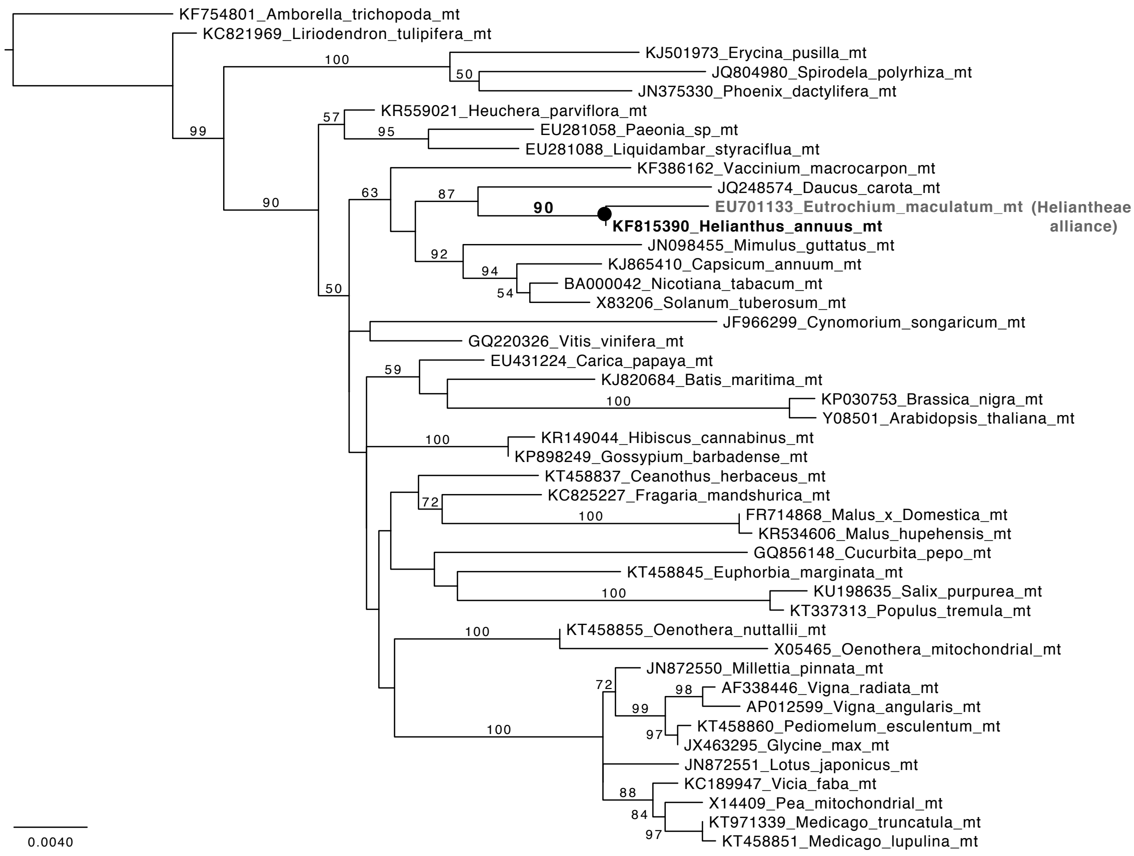

i. *Lophophytum mirabile* (1), *rpl2*, 5’ flanking region, non-coding region (996 nt)

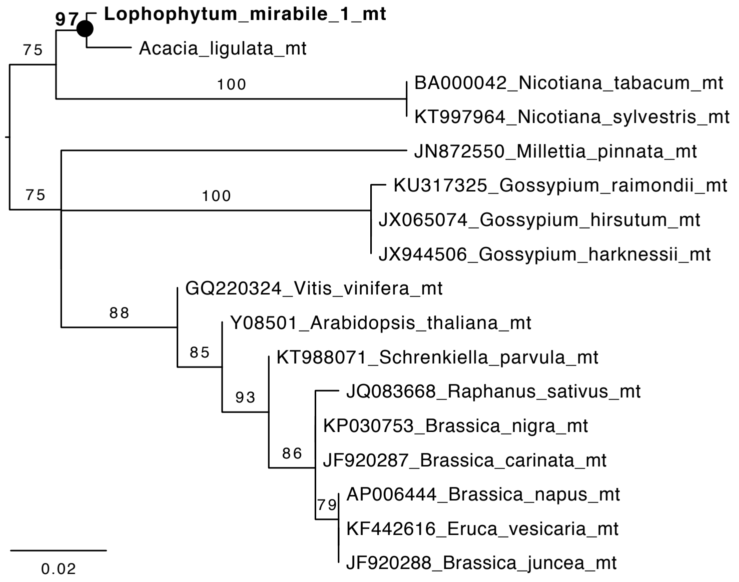

j. *Lophophytum mirabile, rrn23,* 3’ flanking region, non-coding region (811 nt)

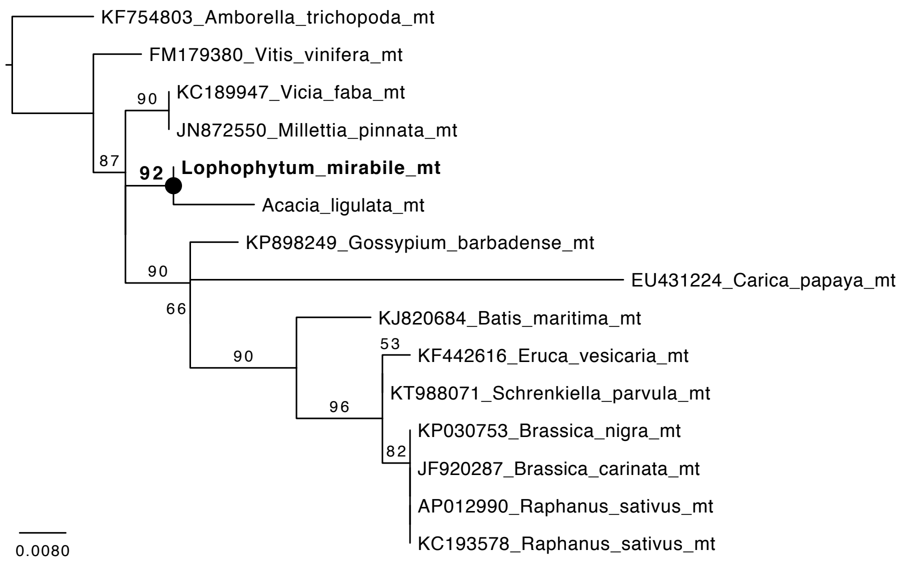

k. *Lophophytum mirabile, psbA,* 3’ flanking region, *nad1* fragment (pseudogene) (1,079 nt)

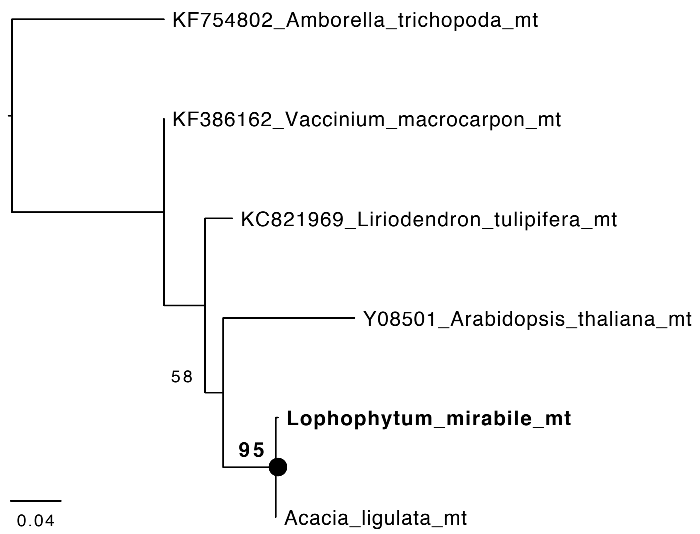

l. *Lophophytum mirabile, petG-trnW,* 5’ flanking region, *nad9* fragment (pseudogene) (385 nt)

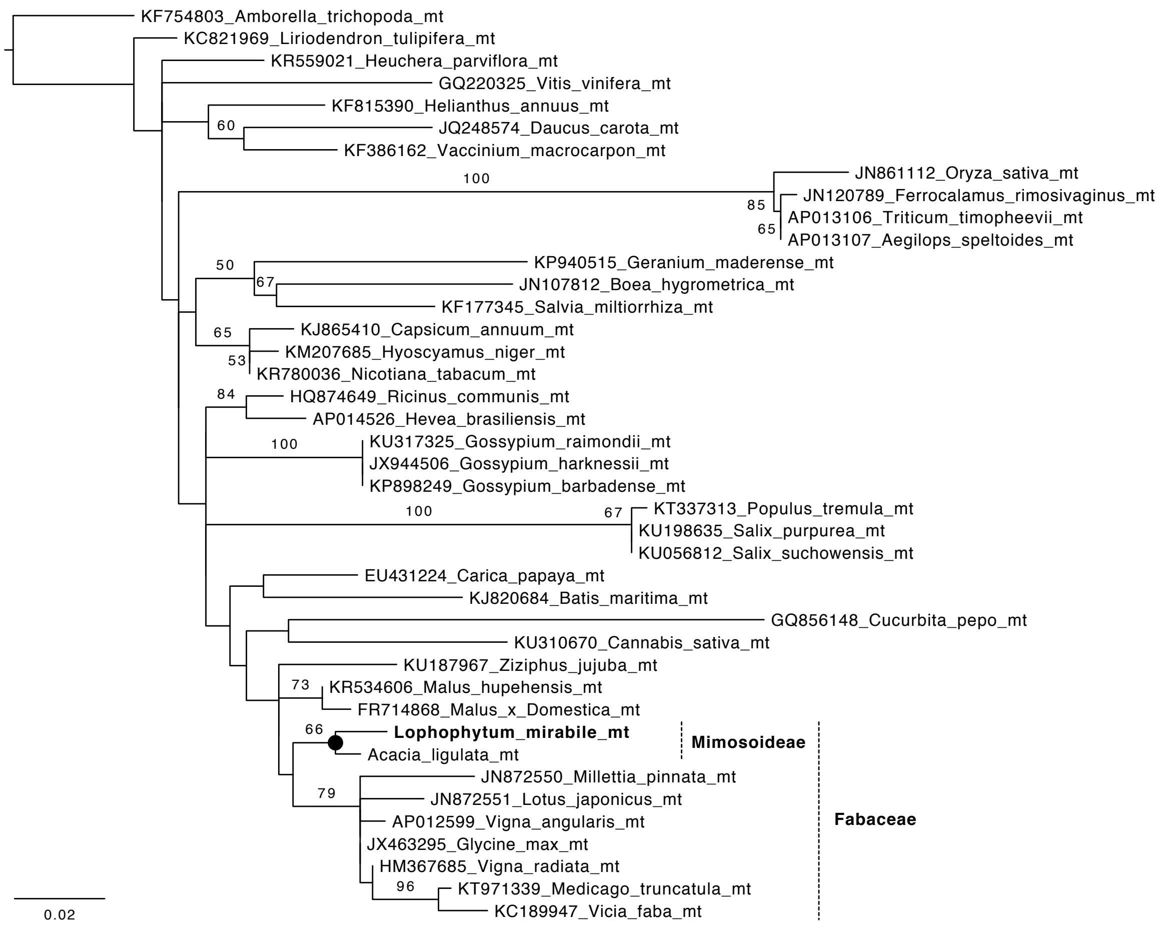

m. *Lophophytum mirabile, rbcL,* 3’ flanking region, non-coding region (1,164 nt)

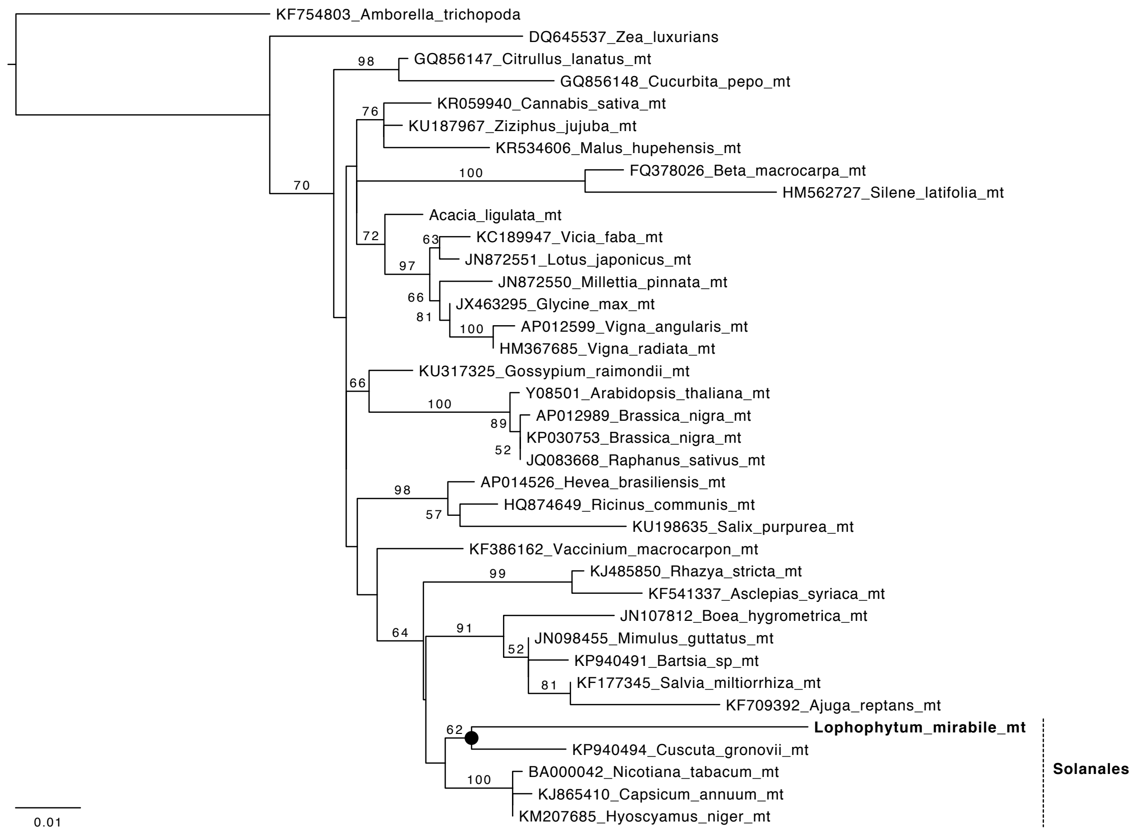

n. *Salvia miltiorrhiza, psbA*, 3’ flanking region, non-coding region (215 nt)

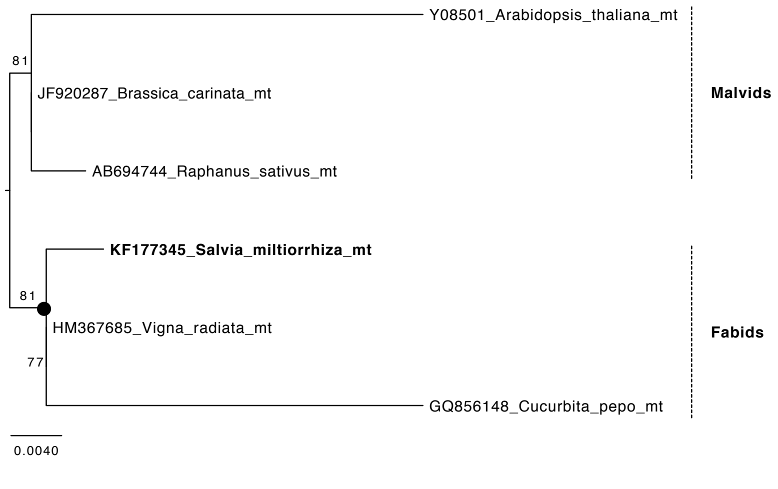

o. *Sapria himalayana*, *psbC-psbD,* 3’ flanking region*, rpl2* fragment (pseudogene) (1,157 nt)

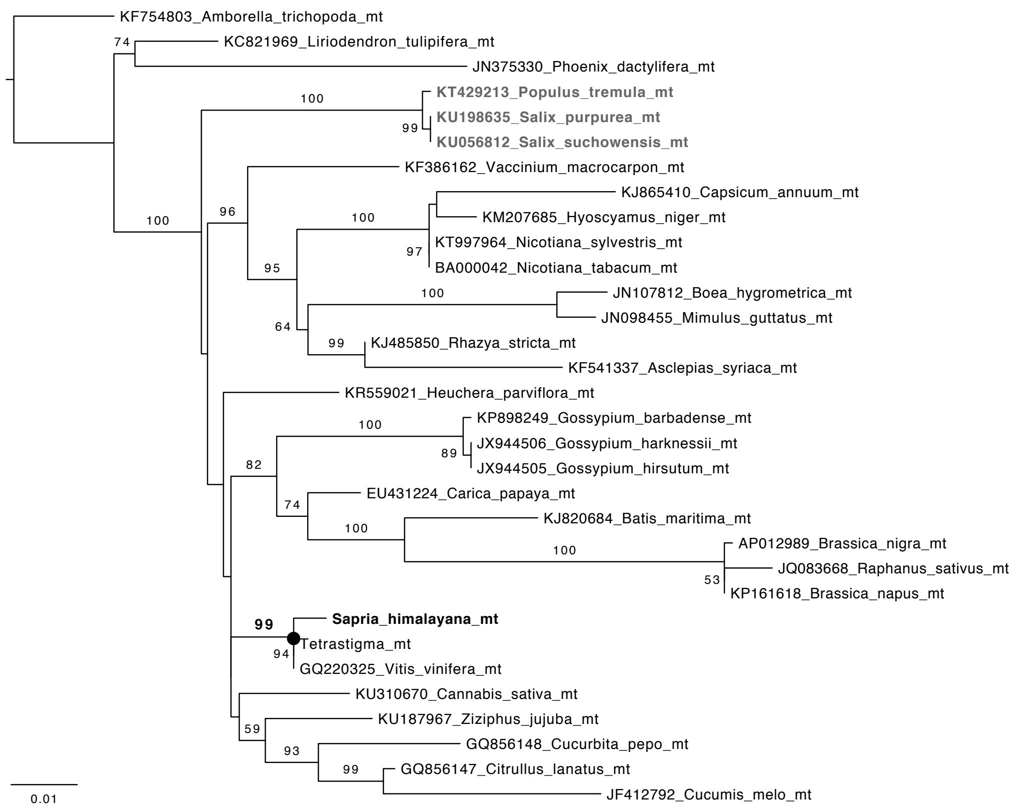

p. *Sapria himalayana*, *atpB,* 3’ flanking region*,* partial *trnQ* (219 nt)

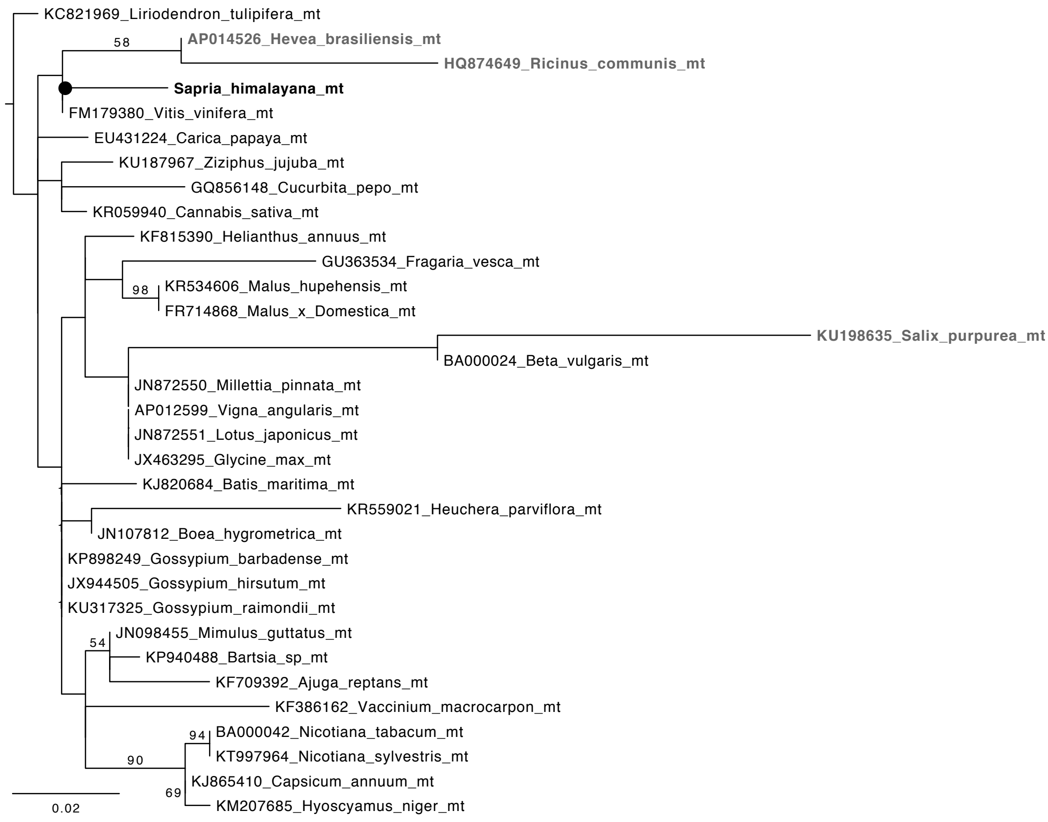

q. *Sapria himalayana*, *rps12,* 3’ flanking region*,* non-coding region (1,048 nt)

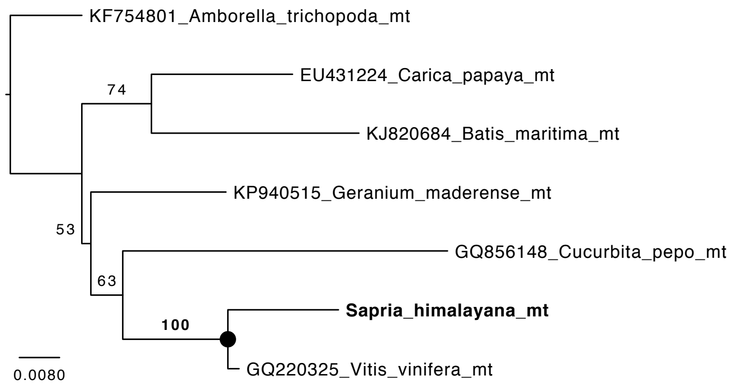

Supplement: Supplementary Information [file srep43402-s1.pdf]
